# Ovalene Photophysics Revisited

**DOI:** 10.1021/acs.jpca.5c08602

**Published:** 2026-02-25

**Authors:** Johannes Wega, Eric Vauthey

**Affiliations:** Department of Physical Chemistry, 27212 University of Geneva, 30 Quai Ernest-Ansermet, 1211 Geneva, Switzerland

## Abstract

Herein, we reinvestigate the photophysics of ovalene, a prototypical nanographene for which conflicting spectroscopic results have been reported. Owing to its structural similarity and its identical *D*_2*h*_ point-group symmetry, ovalene can essentially be viewed as a larger pyrene. We show that its optical transitions can be understood using the same model that is invoked to explain the excited states of pyrene. Absorption and (polarized)-emission measurements reveal that the S_1_ ← S_0_ (^1^B_3u_ ← ^1^A_g_) transition is forbidden, whereas the first prominent absorption band can be assigned to the allowed S_2_ ← S_0_ (^1^B_2u_ ← ^1^A_g_) transition, in contrast to recent reassignments. Temperature and time-dependent spectroscopic measurements show that the S_1_ and S_2_ states quickly establish a thermal pre-equilibrium, giving rise to thermally activated S_2_ → S_0_ emission at room-temperature. As a result, the fluorescence lifetime of ovalene decreases with increasing temperature while its fluorescence quantum yield increases. Contrary to the frequently cited small energy gap of ∼400 cm^–1^, our measurements reveal a significantly larger S_2_–S_1_ gap of approximately 1200 cm^–1^.

## Graphical abstract

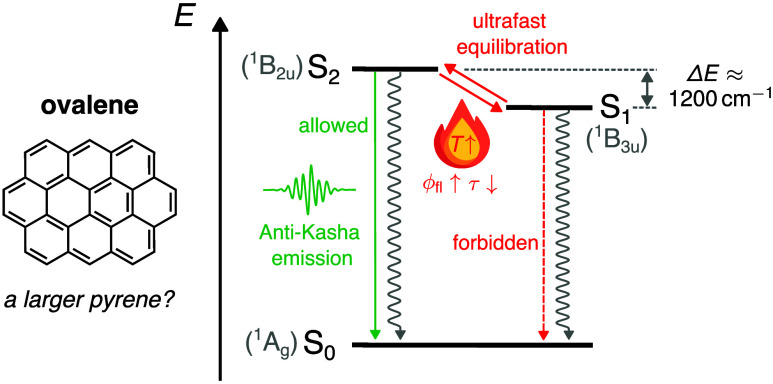

## Introduction

Ovalene (C_32_H_14_) is a large planar polycyclic aromatic hydrocarbon (PAH) of *D*_2*h*_ symmetry (Figure 1A). Owing to its extended π-conjugated system, it has been used as a model system for graphene and is sometimes referred to as a nanographene.^1–5^ Ovalene has also attracted interest as a potential material for nanoelectronics,^6^ quantum dots,^7^ and OLEDs.^8^ Moreover, large PAHs and their radical cations are widely discussed as candidates for emitters in interstellar media,^9,10^ giving ovalene relevance in astrochemistry^10–13^ as well.

**Figure 1:**
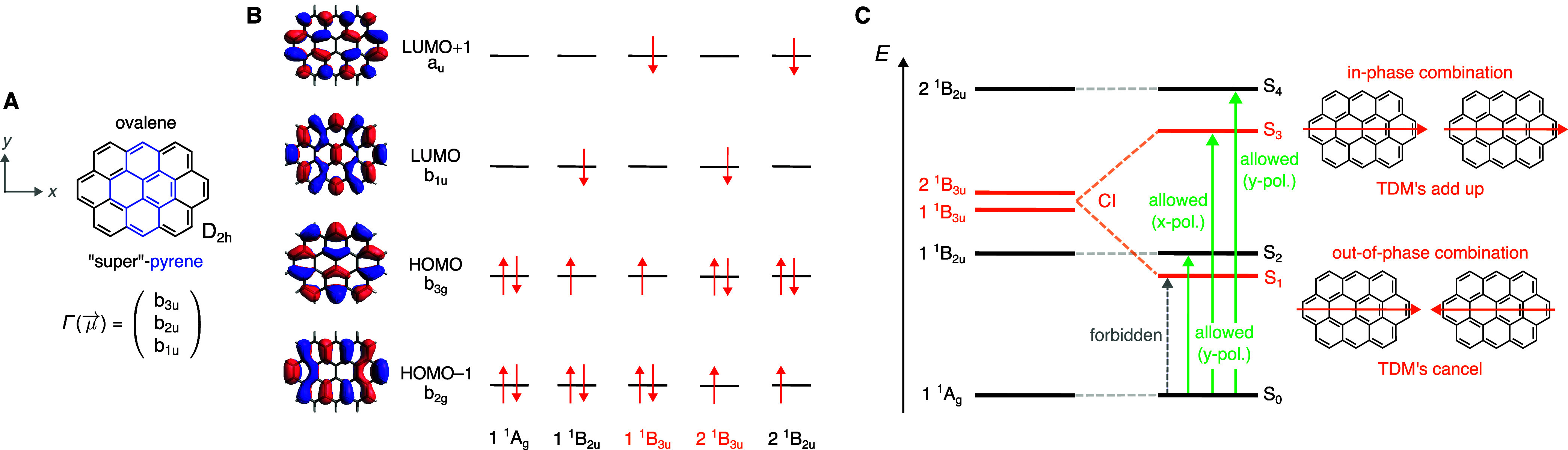
(A) Chemical structure of ovalene together with the molecular coordinate system and the symmetries of the dipole moment operator in the *D*_2*h*_ point group. Ovalene can be considered as a larger pyrene, whose structure is highlighted in blue. (B) Relevant frontier molecular orbitals and electron configurations that are invoked to explain the photophysics of pyrene^28–32^ applied to ovalene. (C) State-energy diagram of ovalene based on the pyrene model.^28–32^ The two near-degenerate B_3u_ states interact by configuration interaction (CI) to form symmetric and antisymmetric combinations. The antisymmetric combination becomes the lowest excited singlet state (S_1_) and is optically dark (S_1_ ← S_0_ forbidden) due to cancellation of the transition dipole moments (TDMs).

Ovalene was first synthesized by Clar in 1948,^14^ who also reported its initial absorption spectrum in 1-methylnaphthalene solution. Clar identified two absorption bands, labeled as *p* and α at 465 and 456 nm, which he tentatively assigned to the S_1_ ← S_0_ and S_2_ ← S_0_ transitions. This wavelength difference corresponds to an S_2_–S_1_ gap of only Δ*E* ≈ 425 cm^–1^ (about 2*k*_B_ *T* at room temperature), suggesting significant thermal population of the S_2_ state even at room temperature. This hypothesis was later confirmed experimentally by Kropp and Stanley in 1971,^15^ who observed clear S_2_ → S_0_ fluorescence in solution and polymer films of ovalene, as well as its disappearance upon cooling down to liquid-nitrogen temperatures. From their temperature-dependent emission measurements, these authors inferred an S_2_–S_1_ energy gap of approximately Δ*E* ≈ 450 cm^–1^, consistent with Clar’s assignment. As a result, this small S_2_–S_1_ gap became widely accepted and entered standard photophysics literature such as John B. Birks famous *Photophysics of Aromatic Molecules* book.^16^

Later on in 1980, Amirav and Jortner investigated the excited-state dynamics of ultracold ovalene in a supersonic jet.^17^ From vibronic excitation and emission spectra, they determined an S_2_–S_1_ energy gap of approximately Δ*E* ≈ 1800 cm^–1^ in the gas phase, i.e., a significantly larger gap than that of ∼450 cm^–1^ proposed earlier. They also reported a fluorescence lifetime of ∼2 μs for cold ovalene, indicating that the S_1_ ← S_0_ transition possesses a very low oscillator strength. This is consistent with the fluorescence lifetimes measured by Kropp and Stanley, who reported a lifetime of ∼400 ns in room-temperature solutions and polymer films.^15^ They also observed a decrease in the fluorescence lifetime by about 200 ns when heating from liquid-nitrogen temperature to room temperature, suggesting an increase in the effective radiative rate due to thermally enhanced population of the S_2_ state with its allowed S_2_ ← S_0_ transition (*vide infra*).

In a follow-up study, Amirav and co-workers examined the solvent dependence of the excited-state lifetime of ovalene.^18^ By increasing the solvent refractive index, they could effectively decrease the S_2_–S_1_ gap. This is because transitions to higher excited states generally exhibit stronger dispersion solvatochromism than those to the first singlet excited state, owing to the higher polarizability of upper-excited states.^19–22^ Therefore, as the S_2_–S_1_ gap decreased, the effective radiative rate was found to increase due to enhanced Herzberg–Teller vibronic coupling between the two transitions. These authors reported an S_2_–S_1_ gap in the range of Δ*E* ≈ 1100–1500 cm^–1^ depending on the refractive index of the solvent. A similar study was also undertaken by Johnson and Offen, who could tune the S_2_–S_1_ gap by varying the pressure of ovalene in a PMMA film.^23^ However, unlike Amirav et al. they reported a gap of 400 cm^–1^ at ambient pressure and temperature in accordance with the measurements of Kropp and Stanley.^15^

More recently, Weber et al. reinvestigated the spectroscopy of ultracold ovalene and reassigned the S_2_ ← S_0_ transition to a ^1^B_3u_ ← ^1^A_g_ transition,^24^ rather than the ^1^B_2u_ ← ^1^A_g_ assignment proposed by Amirav and co-workers.^17^ This conclusion was based on a detailed comparison between their vibrationally resolved excitation and emission spectra and full vibronic Herzberg–Teller TD-DFT simulations of the S_1_ ← S_0_ and S_2_ ← S_0_ transitions.

Here, our aim is to clarify the various discrepancies that persist in the photophysics of ovalene. In particular, our measurements demonstrate that the lowest-energy electronic transitions of ovalene can be fully understood by directly applying the established model used for pyrene to ovalene, owing to the close structural similarity and identical point group of these two molecules. This analysis indicates that the revised symmetry assignment proposed by Weber et al. is inconsistent with experimental evidence. Instead, the transitions should be assigned as S_1_ ← S_0_ (^1^B_3u_ ← ^1^A_g_) and S_2_ ← S_0_ (^1^B_2u_ ← ^1^A_g_), in agreement with earlier works.^17,18^ Furthermore, using temperature-dependent transient absorption spectroscopy, we show that the S_1_ and S_2_ states establish thermal equilibrium on an ultrafast time scale. Additional temperature-dependent emission measurements enable us to determine an energy gap of approximately Δ*E* ≈ 1200 cm^–1^, consistent with the values reported by Amirav and co-workers.^18^

## Methods

### Samples

High purity Ovalene (99%) was purchased from *Kentax GmbH (Germany)* and used without further purification. Spectroscopic grade toluene (TOL, Acros Organics, ≥99.8%), was used as received. Poly­(vinyl butyral) (PVB, B60 H grade) was purchased from *Mowital*.

PVB polymer films of ovalene were prepared by mixing solutions of ovalene and PVB in toluene. The viscous mixture was poured into a glass cylinder positioned on a Petri dish, covered, and left in a fume hood for 1 week to allow for complete solvent evaporation. To detach the films, water was added to the Petri dish and left to stand for several hours. The films were then easily detached from the dish without tearing or sticking, displaying good transparency. Although the PVB films are water-repellent, they were left to dry further in the fume hood for an additional 2 days. The absorbance of the films was below 0.1 at the S_2_ ← S_0_ transition.

### Temperature Control

Temperature-dependent measurements were performed using a *TFC-M25–3* temperature-controlled demountable liquid transmission cell (*Harrick Scientific Products*) equipped with a thermocouple and PID temperature controller. Temperatures between 25 and 150 °C were set using the appropriate PID parameters. Liquid samples were circulated through the cell with a peristaltic pump at a sufficiently low flow rate to ensure thermal equilibration. The cell assembly consisted of a Viton O-ring, 2 mm CaF_2_ window, 950 μm PTFE spacer, sample solution, a second PTFE spacer, a second CaF_2_ window, and a final Viton O-ring. After reaching the set temperature, the system was allowed to equilibrate for several minutes before measurement. For temperature-dependent measurements of PVB films, the film was simply placed between two CaF_2_ windows without spacers.

### Stationary Spectroscopy

Absorption spectra were recorded on a *Cary 50* spectrophotometer. Emission and excitation spectra of ovalene in PVB at room temperature were measured using a *Horiba Nanolog* spectrofluorimeter (*PMT R928*) equipped with a set of polarizers for fluorescence excitation anisotropy measurements. The fluorescence signal was collected in front-face geometry. Raw emission spectra were corrected using a set of secondary emission standards.^25^

Temperature-dependent emission spectra were measured using a home-built setup described in detail in ref 26. Briefly, samples were excited at 355 nm using 2 ns pulses at 10 kHz produced by a laser diode (*MPL-F-355–100 mW, CNI Laser*). The excitation laser was strongly attenuated with neutral density filters to keep fluorescence intensity counts below 1% of the repetition rate to avoid pile-up for TCSPC experiments (see below). The excitation light was focused onto a 30–50 μm spot on the sample. The fluorescence was collected at the front face of the cell at magic angle with respect to the excitation employing a Cassegrain type collection optic (*Anagrain Anaspec Research Laboratories Ltd., Berkshire, U.K.*) in 180° backscattering geometry and was focused into a multimode fiber. The output of the fiber was then guided into a home-built double prism spectrograph,^27^ equipped with a *Pixis 2K* (*Princeton Instruments*) CCD camera. Final emission spectra were corrected using a set of secondary emission standards,^25^ following a pixel-to-wavelength calibration.

During emission measurements, the PVB film was rastered using a micrometer stage to average over multiple locations and mitigate photobleaching. Without rastering, prolonged excitation at a single spot resulted in noticeable spectral changes of the emission spectrum within about 2 min.

### Time-Resolved Fluorescence

Time-resolved fluorescence was measured using a home-built time-correlated single photon counting (TCSPC) setup using the same excitation and collection geometry as the emission spectrograph described above. Instead of coupling the fiber output into the prism spectrograph, the fluorescence was guided into a *Triax-190* (*Horiba*) imaging spectrograph which selected the emission wavelength with a bandpass filter of approximately 3 nm. The spectrally filtered light was then focused onto a photomultiplier (*PMA-C-192-N-M, PicoQuant*), and photon arrival times were recorded with a a *Multiharp 150 N (PicoQuant)*. A fast photodiode placed in the excitation beam path provided the synchronization signal.

At temperatures above 100 °C, the fluorescence decays of ovalene in PVB were purely monoexponential. At lower temperatures, a weak additional fast component appeared, contributing <5% to the total signal. This feature likely originates from a fluorescent impurity with a higher quantum yield than ovalene or from a photodegradation product. As the fluorescence quantum yield of ovalene increases with temperature (*vide infra*), the impurity contribution becomes overshadowed, leading to purely monoexponential decays. Accordingly, decay curves were analyzed either with a monoexponential model or, when necessary at low temperature, with a biexponential decay. In the latter case, only the long-lived component was used for further analysis.

### Transient Absorption Spectroscopy

The transient absorption (TA) setup has been described in detail in ref 33 with the exception that the delay stage has been moved to the probe instead of the pump path. Briefly, the pulses generated by a regeneratively amplified Ti:sapphire system (*Spectra-Physics, Solstice*, 800 nm, 35 fs, 5 kHz) were separated by a beam splitter into pump and probe pulses and were chopped down by mechanical choppers (*Thorlabs*) to 0.5 kHz and 1 kHz, respectively. Pump pulses were generated by an optical parametric amplifier (*TOPAS-Prime* combined with a NirUVis module) tuned to 680 nm, The output of the OPA was frequency doubled using a 100 μm thick β-barium borate (BBO-I, *Castech*) crystal to obtain excitation pulses at 340 nm. The ensuing pump pulses were compressed using a folded prism compressor with two quartz prisms. For probing, a white light supercontinuum (320–750 nm), generated by focusing a fraction of the Solstice output into a 3 mm CaF_2_ plate, was used. Pump and probe pulses were separated in time using a mechanical delay stage (*Physics Instruments*) allowing for a pump–probe delay up to 1.8 ns. The polarization of the pump pulses were set to magic angle with respect to the probe pulses using a set of half wave plates and polarizers. Transient absorption spectra were detected using reference detection using two CCD spectrographs (*Entwicklungsbüro Stresing*, Berlin).^34^

Pump pulses were focused to an approximately 300 μm^2^ spot on the sample. The pump fluence at the sample position was attenuated to roughly 0.3 mJ cm^–2^. Samples were measured in the Harrick cell (see above) under constant flow. Raw TA spectra were corrected for the time-dependent time-zero due to the chirp of the probe light by measuring the optical Kerr effect of the pure solvent as described in ref 35. The average of the background before time-zero was subtracted from the raw spectra. Six scans were averaged. The time resolution of the setup was determined to be around 400 fs to 1 ps (due to the relatively large thickness of the Harrick cell) depending on the probe wavelength by optical Kerr effect measurements in the solvent used for the TA experiments as described in ref 35.

### Quantum-Chemical Calculation

All quantum-chemical calculations were performed using *Gaussian 16*.^36^ The ground-state geometry of ovalene was optimized in the gas phase using density functional theory (DFT) at the B3LYP/6–31G­(d,p) level with imposed *D*_2*h*_ symmetry. All vibration frequencies were positive, indicating that the optimized structure corresponds to an energy minimum. Subsequently, an excited-state calculation of ovalene in vacuum was performed using time-dependent DFT (TD-DFT) at the CAM-B3LYP/6–31G­(d,p) level of theory with the optimized ground-state geometry as input.

## Results and Discussion

### Pyrene Model

Ovalene and pyrene are both PAHs of *D*_2*h*_ symmetry. Comparison of their literature electronic absorption spectra^37^ reveals that ovalene exhibits essentially the same but red-shifted absorption bands as pyrene with nearly identical vibronic progressions (cf. Figure S1), as expected for the larger π-conjugated system of ovalene. This strongly suggests that ovalene and pyrene should display analogous spectroscopic transition patterns.

The underlying electronic transitions of pyrene are well established,^28–32,38^ and Figure 1B,C illustrates the direct transposition of the pyrene model to ovalene. Four excited electron configurations are invoked to explain the photophysics of pyrene (cf. Figure 1B):

1. LUMO ← HOMO (1 ^1^B_2u_)
2. LUMO+1 ← HOMO (1 ^1^B_3u_)
3. LUMO ← HOMO–1 (2 ^1^B_3u_)
4. LUMO+1 ← HOMO–1 (2 ^1^B_2u_)

Symmetry considerations reveal that electronic transitions from the ^1^A_g_ ground state to these excited states are electric-dipole allowed, with the B_3u_ ←A_g_ and B_2u_ ←A_g_ transitions polarized along the *x* and *y* molecular axes, respectively.

In pyrene, however, the S_1_ ← S_0_ transition exhibits a low oscillator strength (ε_max_ ≈ 250 M^–1^ cm^–1^),^37^ consistent with its long fluorescence lifetime of 650 ns in deoxygenated cyclohexane solution.^39^ Quantum-chemical calculations reveal that the two B_3u_ configurations lie close in energy and mix in an almost 50:50 fashion (configuration interaction).^31,32^ The antisymmetric superposition becomes stabilized and falls below the LUMO ← HOMO transition (^1^B_2u_), yielding the spectroscopic S_1_ state (in orange in Figure 1C). Thus, the S_1_ ← S_0_ transition is not symmetry-forbidden per se, but is *accidentally* weak^29^ due to near-complete destructive interference of the transition dipole moments.

Because the mixing is not perfectly 50:50, a small residual transition dipole remains. This allows for experimental observation of the 0–0 band of the S_1_ ← S_0_ transition, which is shown to be *x* polarized as predicted.^29,30^ The S_1_ →S_0_ emission spectrum of pyrene, however, displays both *x*- and *y*-polarized vibronic bands.^29,30^ The *x*-polarized features originate from vibronic transitions involving totally symmetric a_g_ modes, consistent with a pure S_1_ →S_0_ transition. The *y*-polarized peaks arise from Herzberg–Teller coupling between S_1_ ← S_0_ and the nearby allowed S_2_ ← S_0_ transition. For this coupling to occur, the vibration must give the vibronic S_1_ (*v* = 1) state the same symmetry as the S_2_ state, i.e., Γ­(S_1_)⊗Γ­(vib) = Γ­(S_2_) which is only fulfilled with vibrations of b_1g_ symmetry.^29,30^

In addition to multiple experimental indications (see the following sections), several computational arguments also support the application of the pyrene model to ovalene. Quantum-chemical calculations at the time-dependent density functional theory (TD-DFT) level (B3LYP/6–31G­(d,p)//CAM-B3LYP/6–31G­(d,p)) indeed predict the same set of transitions expected from the pyrene model (cf. Table S1, SI), but with an inverted ordering of the S_1_ and S_2_ states. This inversion is a well-known TD-DFT artifact already documented for pyrene,^31,32^ and it can be shown that the correct energy ordering is restored when higher-level methods such as coupled-cluster are employed.^31^ Several experimental observations such as the low oscillator strength of the S_1_ ← S_0_ transition and the long fluorescence lifetime of ovalene (*vide infra*) indicate that the TD-DFT state order must be swapped.

This incorrect TD-DFT ordering also underlies the recent misassignment of the S_2_ ← S_0_ transition as ^1^ B_3u_ ←^1^ A_g_ by Weber et al.^24^ This assignment was deduced by matching the experimental and the TD-DFT Herzberg–Teller vibronic spectra of the computed S_2_ ← S_0_ transition. However, because TD-DFT swaps the states, the calculated spectrum attributed to S_2_ ← S_0_ actually corresponds to the S_1_ ← S_0_ transition. Moreover, the calculated Herzberg–Teller spectrum contains exclusively contributions from a_g_ (*x*-polarized) and b_1g_ (*y*-polarized) vibrations (cf. Table S3 in ref 24), i.e., precisely the symmetry pattern observed experimentally in the S_1_ →S_0_ emission band of pyrene.

Contrary to the pyrene model, the TD-DFT calculations for ovalene predict symmetry-forbidden dark states of *gerade* (g) symmetry above the S_2_ state (cf. Table S1, SI). These states may indeed exist, however, for clarity and consistency, we will label the experimental spectra according to the pyrene model.

### Absorption Spectrum

Figure 2A shows the stationary electronic absorption spectrum of ovalene in toluene. In a conventional 1 cm cell, the first observable absorption band appears around 450 nm with extinction coefficients of the order of 10^4^ M^–1^cm^–1^. This strong absorption points to an allowed transition, which cannot correspond to the S_1_ ←S_0_ transition according to the pyrene model. We thus assign this feature to the allowed S_2_ ←S_0_ transition. At higher energy, around 340 nm, an even stronger absorption band is observed, which we assign to the S_3_ ←S_0_ transition, consistent with the pyrene model and its large extinction coefficients (∼10^5^ M^–1^cm^–1^) arising from the symmetric combination of the two *x*-polarized transition dipoles.

**Figure 2:**
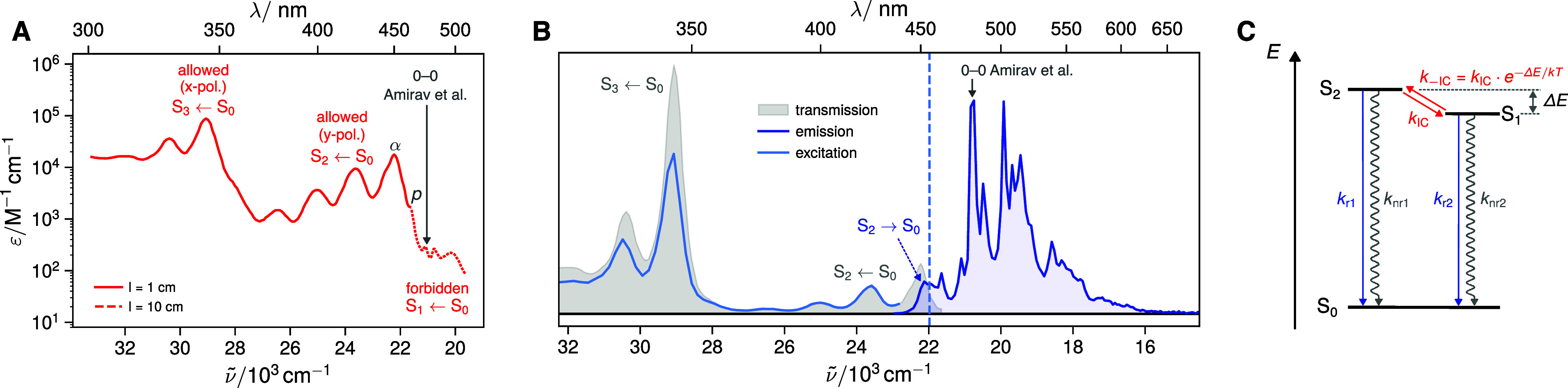
(A) Absorption spectrum of ovalene (*c* = 13 μM) in toluene measured in a 1 cm (solid line) and 10 cm (dashed line above ∼460 nm) cell. The absorption bands are labeled according to their assignments based on the pyrene model (cf. Figure 1). The historic α and *p* band assignment according to Clar is also shown.^14^ Using these bands as the 0–0 origins of the S_2_ ← S_0_ and S_1_ ← S_0_ transitions would imply a S_2_–S_1_ gap of Δ*E* = 400 cm^–1^. The origin of the S_1_ ← S_0_ transition proposed by Amirav et al.,^17,18^ suggesting instead a significantly larger energy gap around Δ*E* = 1200 cm^–1^ is also highlighted. (B) Emission (blue, excitation at 420 nm), excitation (light blue, emission at 455 nm, blue dashed vertical line), and transmission (1 – 10^–A^, gray) spectra of ovalene in a polyvinyl butyral (PVB) film, demonstrating emission from the S_2_ state. (C) Photophysical model used to rationalize the spectroscopy of ovalene: rapid equilibration between the populations of S_2_ and S_1_ states by fast internal conversion (*k*_IC_) and thermally activated back-IC (*k*_–IC_) occurs prior to radiative (*k*_r_) or nonradiative (*k*_nr_) decay to the ground state.

The figure also shows the historic α and *p* band assignment of Clar,^14^ which suggests a small S_2_–S_1_ gap of Δ*E* ≈ 400 cm^–1^. However, the absorption spectrum measured in a 10 cm cell reveals additional bands above 460 nm with extinction coefficients of the order of 100 M^–1^cm^–1^. This is closely analogous to the absorption spectrum of pyrene (Figure S1) and we therefore assign these weak bands to the S_1_ ←S_0_ transition, which is missed in the published absorption spectra of ovalene in solution.^37^ Using the α-band origin and the S_2_–S_1_ gap proposed by Amirav and co-workers of around Δ*E* ≈ 1200 cm^–1^, one ends up on 474 nm peak, which therefore may correspond to the 0–0 origin of the S_1_ ←S_0_ transition.

The assignment of the absorption bands according to the pyrene model is further supported by comparing the estimated oscillator strengths, *f*, and radiative rate constants, *k*_r_, obtained by integration of the respective absorption bands using the Strickler–Berg relation (Figure S2). This yields *f* = 0.002 and *k*_r1_ = 10^6^ s^–1^ for S_1_ ←S_0_, and *f* = 0.10 and *k*_r2_ = 7 × 10^7^ s^–1^ for S_2_ ←S_0_. Comparison of these values with the TD-DFT calculations (Table S1) further corroborates this assignment.

### Emission and Excitation Spectra

Figure 2B shows the emission spectrum of ovalene in toluene at room temperature upon excitation in the S_2_ ← S_0_ band at 420 nm (dark blue). The spectrum exhibits a complicated vibronic structure characteristic of a forbidden transition and resembles the emission spectrum of pyrene.^40^ However, additional emission features appear significantly blue-shifted from the 0–0 origin of the S_1_ →S_0_ transition proposed by Amirav and co-workers.^17,18^ Most strikingly, a broad band is observed around 450 nm, with a shape resembling the mirror image of the first band in the vibronic progression of the S_2_ ← S_0_ transition.

An excitation spectrum recorded within this band at 455 nm matches the transmission spectrum of ovalene, confirming that this emission originates from ovalene rather than from an impurity. It can therefore be assigned to anti-Kasha S_2_ →S_0_ fluorescence.

Kropp and Stanley reported that the S_2_ →S_0_ emission band completely disappears upon cooling to liquid–nitrogen temperature,^15^ and interpreted the photophysics of ovalene in terms of the scheme shown in Figure 2C. This model assumes that the populations of the S_2_ and S_1_ states rapidly establish a thermal pre-equilibration through internal conversion (IC) with a rate constant *k*_IC_ and thermally activated back-IC with *k*_– IC_ = *k*_IC_·e^–Δ*E*/*kT*^ before either excited state deactivates radiatively (with rate constants *k*_r1_ and *k*_r2_) or nonradiatively (with rate constants *k*_nr1_ and *k*_nr2_) to the ground state. The assumption of rapid equilibration is well justified because S_2_ → S_1_ internal conversion typically occurs on a subpicosecond time scale. For example, in pyrene an S_2_ →S_1_ IC time constant of about 150–300 fs has been reported.^41–43^

The observation of anti-Kasha emission for ovalene but not for pyrene points a significantly smaller S_2_–S_1_ energy gap in ovalene compared to pyrene such that the S_2_ state can be populated even at room temperature. For comparison, the S_2_–S_1_ gap in pyrene is reported to be approximately 3000 cm^–1^.^16^

What are the consequences of such S_2_–S_1_ equilibration (Figure 2C) for the photophysics of ovalene? As derived in detail in Section S3, this model predicts that the concentrations of molecules in the equilibrated S_1_ and S_2_ states, after optical excitation in the S_2_ ← S_0_ band, are given by

1 $${\left[S_{1}\right]}_{\mathrm{eq}}={\left[S_{2}\right]}_{0}\cdot \frac{1}{1+e^{-\Delta E/k_{B}T}}$$

2 $${\left[S_{2}\right]}_{\mathrm{eq}}={\left[S_{2}\right]}_{0}\cdot \frac{e^{-\Delta E/k_{B}T}}{1+e^{-\Delta E/k_{B}T}}$$

with [S_2_]_0_ denoting the initial concentration of molecules in S_2_ at *t* = 0. Once equilibrium established, the excited-state populations decay monoexponentially with an effective rate constant

3 keff(T)=[S1]eq[S2]0·(kr1+knr1)+[S2]eq[S2]0·(kr2+knr2)=kr1+knr11+e−ΔE/kBT+kr2+knr21+e+ΔE/kBT

where the decay rates of the two individual states are weighted by their relative equilibrium populations. The model therefore predicts a decrease in the fluorescence lifetime of ovalene with increasing temperature, since as the thermal population of S_2_ increases, the excited-state population deactivates more efficiently via the allowed S_2_ → S_0_ transition. Consequently, the larger effective radiative rate at higher temperature implies an increase in the fluorescence quantum yield (Figure S4).

Using temperature-dependent emission measurements, Kropp and Stanley inferred an S_2_–S_1_ energy gap of Δ*E* ≈ 450 cm^–1^.^15^ We will later show that their procedure for extracting this value was flawed, and that the temperature-dependent emission measurements in fact reveal an energy gap close to Δ*E* ≈ 1200 cm^–1^, in agreement with the value reported by Amirav and co-workers.^18^

Another indication that Amirav’s energy gap value is correct emerges from the comparison of the Herzberg–Teller–computed TD-DFT emission spectrum reported by Weber et al.^24^ with the experimental one. Qualitatively good agreement between experiment and theory is only obtained when the 0–0 transition is positioned according to Amirav’s value (Figure S8A). In contrast, when the emission origin is placed at the position of the *p*-band, the calculated and experimental spectra do no longer coincide (Figure S8B).

It is remarkable that emission from the S_2_ state of ovalene is observed at all, given that its thermal population is expected to be below 1% at room temperature (Figure S3). The reason for S_2_ emission to be visible lies in the much larger radiative rate constant *k*_r2_ compared to *k*_r1_.

### Fluorescence Excitation Anisotropy

In an attempt to verify the polarization of the absorption bands of ovalene predicted by the pyrene model, we performed polarization-resolved emission spectroscopy with ovalene embedded in a rigid poly­(vinyl butyral) (PVB) film. Figure 3 shows the fluorescence excitation anisotropy, *r*, measured at two different emission wavelengths: 452 nm (blue dots), within the S_2_ →S_0_ emission band, and 481 nm (red dots), close to the S_1_(*v* = 0)→ S_0_(*v* = 0) transition as assigned by Amirav and co-workers.^17,18^

**Figure 3:**
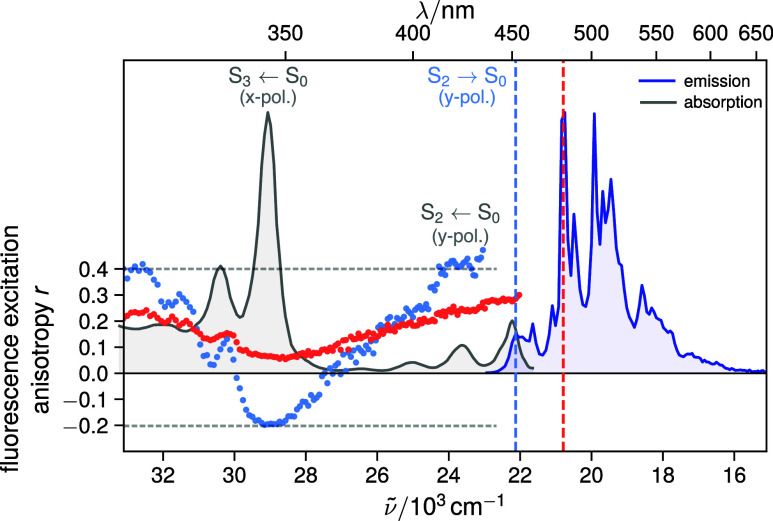
Fluorescence excitation anisotropy of ovalene in a PVB film measured at an emission wavelength of 452 nm (blue dots) within the S_2_ → S_0_ band (blue dots, the >0.4 values above 430 nm is due to the contribution of scattered excitation light) and at 481 nm (red dots) near the S_1_(*v* = 0)→ S_0_(*v* = 0) transition. The absorption and emission spectra are shown for comparison as gray and blue shaded areas, respectively. The vertical dashed lines represent the wavelengths at which fluorescence anisotropy was measured. The data clearly demonstrates that S_2_ emission is *y*-polarized in complete accordance with the pyrene model. Emission from the forbidden S_1_ state shows both *x*- and *y*-polarized vibronic features (see text), yielding an anisotropy spectrum that varies with emission wavelength.

We first consider the anisotropy measured in the S_2_ →S_0_ emission band. According to the pyrene model (Figure 1C), S_2_ emission is expected to be *y*-polarized. Excitation in the S_2_ ← S_0_ absorption band should yield an anisotropy of *r* = +0.4 (parallel transition dipole moments for excitation and emission). Furthermore, the pyrene model predicts that the higher-energy absorption band below 350 nm, assigned to the S_3_ ←S_0_ transition, is *x*-polarized. Thus, an S_2_ fluorescence anisotropy of *r* = −0.2 is expected upon excitation in the S_3_ ←S_0_ band.

The experimental data confirm these predictions remarkably well, i.e., the fluorescence excitation anisotropy decreases from *r* = +0.4 in the S_2_ ← S_0_ band to *r* = −0.2 at the origin of the S_3_ ←S_0_ transition (Figure 3), exactly as expected from the pyrene model. Upon moving further into the UV beyond the S_3_ ←S_0_ origin, the anisotropy again approaches a value of *r* = +0.4. This behavior is also observed in analogous measurements with pyrene, where it has been shown that the higher-energy side of the S_3_ ←S_0_ band already contains contributions from the *y*-polarized S_4_ ←S_0_ transition.^29,30^

We now move to the fluorescence excitation anisotropy measured near the S_1_(*v* = 0) → S_0_(*v* = 0) transition (red dots). The pyrene model predicts this emission to be purely *x*-polarized. Consequently, one would expect an anisotropy of *r* = −0.2 in the S_2_ ← S_0_ band and *r* = +0.4 in the S_3_ ←S_0_ band. However, as discussed above, the S_1_ →S_0_ emission contains both *x*-polarized (a_*g*_) and *y*-polarized (b_1g_, via Herzberg–Teller coupling to the S_2_ state) vibronic bands. Indeed, the Herzberg–Teller TD-DFT spectrum calculated by Weber et al. (Figure S9) shows that the *x*- and *y*-polarized vibronic transitions overlap substantially once the spectrum is broadened in solution. As a result, the observed emission in the S_1_ →S_0_ region generally contains contributions of both polarization directions, with different relative weights depending on the emission wavelength. Furthermore, additional *y*-polarized vibronic features of the S_2_ →S_0_ emission that likely lie underneath the S_1_ →S_0_ band further increase the overall *y*-polarized character of the emission. For an emission that is 50% *y*-polarized and 50% *x*-polarized, one would expect an anisotropy of *r* = +0.1 in both the S_2_ ← S_0_ and S_3_ ←S_0_ absorption bands.

The experimental anisotropy shows that although the S_1_ emission still contains significant *y*-polarization, likely due to underlying *y*-polarized Herzberg–Teller active modes or residual S_2_ emission at this wavelength, it nevertheless acquires a substantial amount of *x*-polarized character compared to the anisotropy measured within the S_2_ →S_0_ band, further supporting our spectroscopic assignments.

### Transient Absorption Spectroscopy

Figure 4 depicts transient absorption (TA) spectra measured at different temperatures 2 ps after S_3_ ← S_0_ excitation of ovalene in toluene. All spectra exhibit a negative signal below 460 nm that matches the S_2_ ← S_0_ absorption band and is thus assigned to the ground-state bleach (GSB). The positive bands present above 460 nm are attributed to excited-state absorption (ESA), which may originate from either the S_1_ or S_2_ states (*vide infra*).

**Figure 4:**
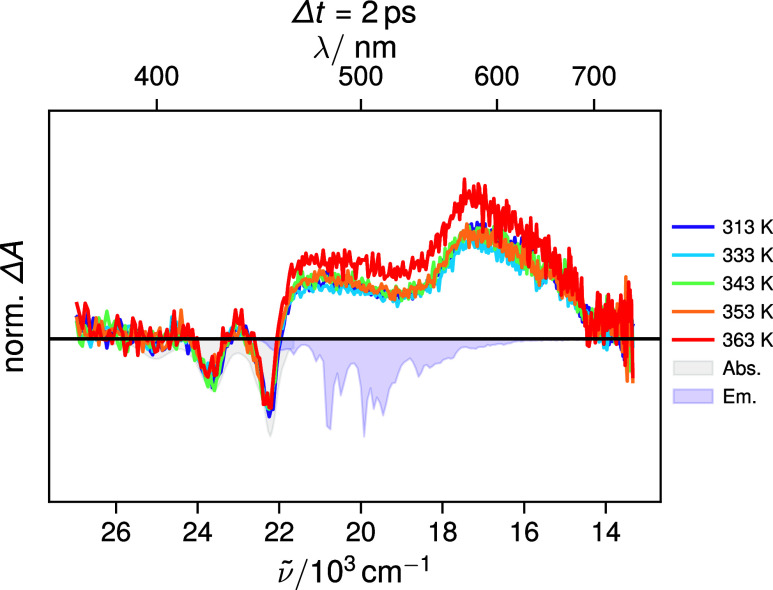
Transient absorption spectra measured at different temperatures 2 ps after S_3_ ← S_0_ excitation at 340 nm of ovalene in toluene. All spectra were normalized to the integral over the ground-state bleach region. The absolute signal of the bleach at 450 nm was around 0.7 mOD. The stationary absorption and emission spectra are shown as blue and gray shaded areas, respectively.

No significant temperature-dependent changes in either the spectral dynamics or overall band shape were detected within the experimental time window (Figure S10). The spectral profile shown in Figure 4 is present from the earliest measurable time delay and remains essentially unchanged over the 1.8 ns time window of the experiment.

These results indicate that the equilibration between the S_2_ and S_1_ states illustrated in Figure 2C is ultrafast and occurs entirely within the instrument response function of the experiment. Consequently, the first observable TA spectrum already reflects an equilibrium mixture of the S_2_ and S_1_ populations. Because the S_1_ ← S_0_ transition is intrinsically forbidden, the excited-state population exhibits a long lifetime (see time-resolved emission measurements below) and therefore does not decay appreciably on the sub-10 ns time scale. As discussed in the ‘pyrene model’ section, the S_1_ ← S_0_ transition of pyrene is also forbidden. Like for ovalene, its TA spectrum remains essentially unchanged on the hundreds of picosecond time scale, due to its long S_1_ state lifetime of several hundreds of nanoseconds.^41,42^

Ideally, if the S_2_ ⇌ S_1_ equilibration could be time-resolved, one should initially observe a strong negative stimulated emission (SE) signal from the S_2_ → S_0_ transition, with a magnitude comparable to the ground-state bleach (GSB), since all excited population initially resides in the S_2_ state. As equilibration proceeds, this SE signal should rapidly vanish. For an energy gap of Δ*E* = 1200 cm^–1^ only about 1% of the total excited-state population remains in the S_2_ state at equilibrium (Figure S3). The effective radiative rate is then dominated by the much smaller radiative rate of the forbidden S_1_ → S_0_ transition, which would yield a SE contribution similar in magnitude to the weak S_1_ ← S_0_ absorption band and would thus be likely undetectable in TA, especially given the small signal amplitudes obtained for ovalene due to its limited solubility in organic solvents (Figure 3).

Alternatively, if the S_*n*_ ←S_2_ and S_*n*_ ←S_1_ ESA features had comparable extinction coefficients, their relative amplitudes after equilibration should directly reflect Δ*E* (cf. eqs 1 and 2). However, at equilibrium, at most 1% of the overall excited population should be in the S_2_ state at room temperature. Therefore, no significant change in the TA signal is anticipated upon varying temperature. Considering that the amplitude of the signal is of the order of 1 mOD, the expected change is well below the noise level of the experiment. The slight apparent increase in signal intensity at 363 K likely falls within experimental uncertainty. Furthermore, temperature-induced changes in the band shapes of the observed transitions may also contribute.

Although the S_2_ ⇌ S_1_ equilibration could not be time-resolved, the TA data nonetheless indicate that this process is ultrafast, as expected for internal conversion between close-lying electronic states. Such small gap favors a large overlap of the vibrational wave functions of the two electronic states and thus ultrafast IC, as is well-known from the energy-gap law.^44,45^

### Temperature-Dependent Emission Spectra

We now turn to the temperature dependence of the stationary emission spectrum, which, unlike the equilibrated TA spectra, is expected to be much more pronounced (see Section S3.3). Figure 5 shows the emission spectra of ovalene in a PVB film measured between 298 to 413 K in 5 K steps. As predicted by the photophysical model in Figure 2C, the intensity of the S_2_ → S_0_ band, mostly visible near 450 nm, increases with temperature. Simultaneously, the sharp vibronic peaks associated with the forbidden S_1_ → S_0_ transition above 460 nm lose intensity, and the overall spectrum becomes increasingly broadened. The experimental temperature-dependent spectra are in excellent qualitative agreement with simulations based on the photophysical model assuming an energy gap of Δ*E* = 1200 cm^–1^ (Figure S6).

**Figure 5:**
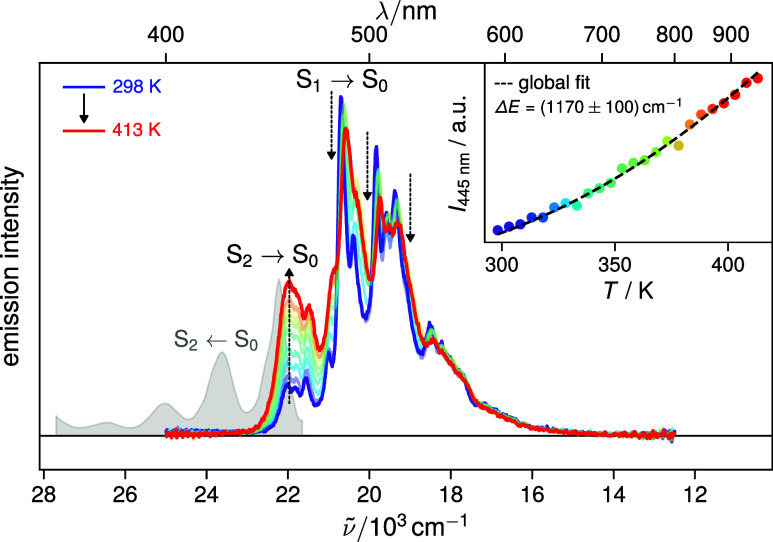
Emission spectra measured with ovalene in a PVB film between 298 and 413 K illustrating the increase of S_2_ → S_0_ emission and the concurrent decrease of S_1_ → S_0_ emission, consistent with the photophysical model in Figure 2C. The inset depicts a fit of eq 4 to the emission intensity at 445 nm in the S_2_ → S_0_ band (see also Figure S14). All wavelengths between 443–450 nm were analyzed globally using a single S_2_–S_1_ energy gap Δ*E* (Figure S12). These temperature-dependent emission spectra are in good agreement with simulations based on the photophysical model with the extracted energy gap of Δ*E* ≈ 1200 cm^–1^ (Figure S6).

As derived in detail in Section S3.3, the fluorescence intensity *F*_2_ at a wavelength within the S_2_ → S_0_ band, without any contribution from S_1_ emission, should follow a simple Boltzmann-type dependence

4 $$F_{2}\left(\lambda ,T\right)\approx C\left(\lambda \right)e^{-\Delta E/k_{B}T}$$

where *C*(λ) is an emission wavelength dependent constant. To extract the S_2_–S_1_ gap from the temperature-dependent spectra, we globally analyzed 38 wavelengths located on the red edge of the S_2_ →*S*_0_ emission band, to avoid potential overlap with S_1_ emission, using eq 4 with one common Δ*E* value (Figure S12). One representative fit at 445 nm is shown in the inset of Figure 5. The resulting value of Δ*E* ≈ 1200 cm^–1^ is in excellent agreement with that reported by Amirav and co-workers.^18^

Stanley and Kropp extracted a value of Δ*E* = 450 cm^–1^ from similar measurements using the same eq 4.^15^ What is then the origin of this discrepancy in the Δ*E* value ? The key point is that eq 4 is only valid when the monitored emission arises exclusively from the S_2_ → S_0_ transition. Stanley and Kropp analyzed the emission intensity at single wavelength only, namely 475 nm, where both S_2_ and S_1_ emissions contribute. In this case, the temperature dependence of the fluorescence intensity does no longer follow a simple Boltzmann law (eq S19). Indeed, when analyzing the 475 nm data only, we also find an apparently small gap close to 400 cm^–1^ (Figure S11).

For eq 4 to be applicable, it must also be assumed that the intrinsic lineshapes of the S_1_ → S_0_ and S_2_ → S_0_ transitions do not change significantly with temperature. While this assumption may be reasonable for the allowed S_2_ → S_0_ transition, forbidden transitions like the S_1_ → S_0_ transition often exhibits complicated temperature-dependent lineshapes due to variations of vibronic coupling with temperature.^46^ Consequently, analyzing a wavelength within the S_1_ → S_0_ band is problematic.

Stanley and Kropp selected the emission at 475 nm for their analysis because they found that this vibronic feature disappears entirely upon cooling down to 133 K and concluded that it originates from S_2_ → S_0_ emission.^15^ However, Amirav’s assignment and Weber’s TD-DFT calculations indicate that the true 0–0 transition is more likely the peak near 480 nm (Figure S7).^17,24^ The 475 nm feature may therefore originate from a vibrationally hot S_1_(*v*≠0) → S_0_(*v* = 0) transition, which would likewise explain its disappearance at low temperature.

### Temperature Dependent Time-Resolved Emission

As discussed above, the photophysical model in Figure 2C predicts a decrease of the fluorescence lifetime of ovalene with temperature and an increase of its fluorescence quantum yield (cf. Figure S4). We now turn to the experimental verification of these predictions. Figure 6 A shows the time-resolved emission of ovalene in PVB at 470 nm upon 355 nm excitation, recorded at room temperature (298 K, purple) and at 423 K (red) using the same integration time. At 298 K, the kinetics are well described by a monoexponential decay with a lifetime of ∼380 ns, consistent with deactivation being dominated by the slow radiative rate of the forbidden S_1_ → S_0_ transition. Upon heating to 423 K, the lifetime decreases by nearly a factor of 2 to ∼180 ns. This reduction reflects the increase in the effective deactivation rate *k*_eff_ of the excited-state equilibrium population (cf. eq 3) as thermal population of the S_2_ state is increased and the allowed S_2_ → S_0_ radiative pathway contributes more strongly. Simultaneously, the time-integrated decay, which is proportional to the fluorescence quantum yield, increases upon heating as expected from the model.

**Figure 6:**
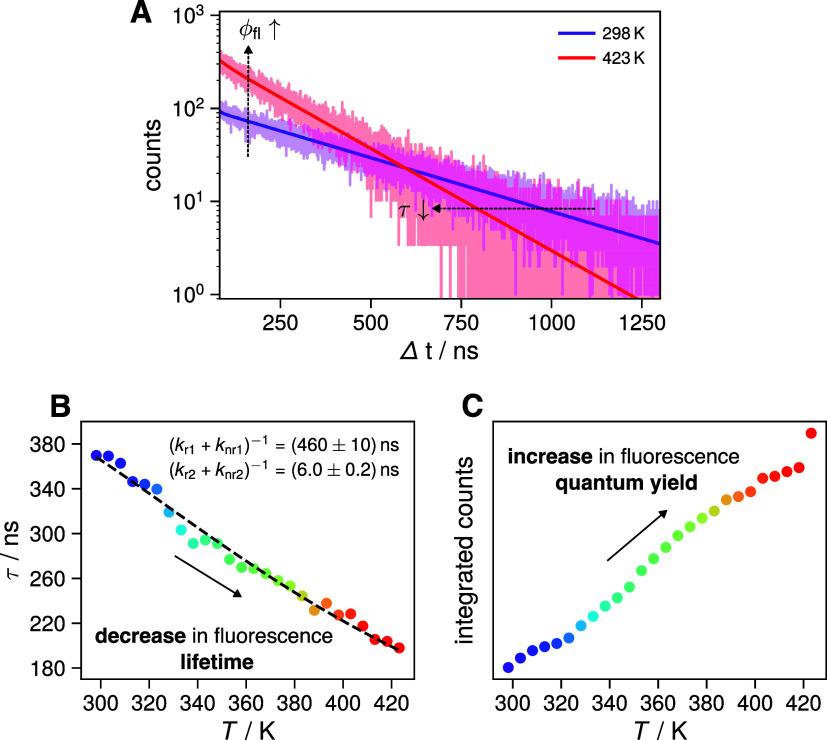
(A) Representative fluorescence time profiles measured at 470 nm with the same integration time after 355 nm excitation of ovalene in a PVB film at 298 K (purple) and 423 K (red). (B) Extracted monoexponential fluorescence lifetimes as a function of temperature (dots) together with a nonlinear least-squares fit of eq 3 to τ­(*T*) = 1/*k*_eff_(*T*), using Δ*E* = 1200 cm^–1^ and τ_1_ and τ_2_ as adjustable parameters. (C) Time-integrated fluorescence decay as a function of temperature.

To test whether these trends are general, we measured the emission decay between 298 and 423 K in 5 K steps. As illustrated in Figure 6B, the fluorescence lifetime decreases continuously with temperature, while the time-integrated fluorescence (Figure 6C) increases accordingly.

To evaluate whether the temperature-dependent lifetimes are consistent with the photophysical model in Figure 2C, we fitted the expression τ = 1/*k*_eff_ (using eq 3 for *k*_eff_) to the measured lifetimes with a fixed energy gap of Δ*E* = 1200 cm^–1^ and with τ_1_ = (*k*_r1_ + *k*_nr1_)^−1^ and τ_2_ = (*k*_r2_ + *k*_nr2_)^−1^ as adjustable parameters (Figure 6B). The time constants τ_1_ and τ_2_ can be thought of as the fluorescence lifetimes of the S_1_ and S_2_ states if internal conversion between them would be disabled. The extracted values, τ_1_ ≈ 450 ns and τ_2_ ≈ 6 ns, are in excellent agreement with the expected characters of the two transitions, i.e., S_1_ → S_0_ being forbidden and S_2_ → S_0_ being allowed and thus with their corresponding radiative rate constants. These results further confirm that the scheme in Figure 2C accurately captures the photophysics of ovalene. Using different Δ*E* values does not lead to a good fit to the data as illustrated in Figures S13 and S14. Similarly, using different Δ*E*, while using fixed τ_1_ and τ_2_ values, result in bad fits as well.

## Conclusions

Many contradictory statements regarding the photophysics of ovalene persist in the literature. In this work, we presented a systematic spectroscopic reinvestigation of the excited-state properties of ovalene. Our measurements demonstrate that ovalene exhibits essentially the same pattern of electronic transitions as its smaller analogue, pyrene, i.e., an accidentally forbidden S_1_ ← S_0_ transition arising from an out-of-phase combination of two coupled transition dipole moments, and a fully allowed S_2_ ← S_0_ transition. Polarization-dependent measurements further show that these bands correspond to ^1^B_3u_ ← ^1^A_g_ and ^1^B_2u_ ← ^1^A_g_ transitions respectively, in contrast to the recent reassignment proposed by Weber et al.^24^

Unlike pyrene, however, ovalene possesses a significantly smaller S_2_–S_1_ energy gap. Our temperature- and time-dependent measurements indicate that the photophysics of ovalene is accurately described by the model presented in Figure 2C, which assumes an ultrafast equilibration between the S_2_ and S_1_ states via internal conversion and thermally activated back-internal conversion prior to radiative or nonradiative decay from either state.

Contrary to the frequently used value of Δ*E* ≈ 400 cm^–1^,^15,16,23^ our measurements reveal a significantly larger gap of Δ*E* ≈ 1200 cm^–1^ in a PVB film. This value is consistent with earlier reports by Amirav and co-workers,^17,18^ who observed S_2_–S_1_ gaps ranging from 1100 to 1500 cm^–1^ depending on the refractive index of the surrounding medium. We show that the value of Δ*E* ≈ 400 cm^–1^ results from the analysis of the S_2_ → S_0_ emission at a wavelength where S_1_ → S_0_ contributions are significant, from earlier misassignments of the S_1_ ← S_0_ 0–0 transition and from temperature-dependent S_2_ → S_0_ line-shape effects.

A consequence of the photophysical model is that the S_2_ state can be thermally populated from the long-lived S_1_ state, which allows for the observation of anti-Kasha S_2_ → S_0_ emission of ovalene even at room temperature. Moreover, as the temperature increases, we find that the fluorescence lifetime of ovalene decreases continuously, while its fluorescence quantum yield and S_2_ → S_0_ emission intensity increase. Both trends arise from the increasing thermal population of S_2_, which enhances the contribution of the fast radiative decay of the allowed S_2_ → S_0_ transition to the overall deactivation of the excited-state equilibrium population relative to the much slower radiative rate of the forbidden S_1_ → S_0_ transition. These properties may make ovalene a potentially interesting fluorescent temperature probe.

## Supplementary Material



## Notes

Experimental and computational details. Comparison between pyrene and ovalene absorption spectra, TD-DFT calculations, simulations and expectations from the photophysical model including derivations of key equations, comparison between calculated and experimental emission spectra, additional transient absorption data, temperature dependent emission at 475 nm and global fit results (PDF)
