# Supplementary material for: Ovalene Photophysics Revisited
Source: J Phys Chem A. 2026 Feb 25;130(10):2148–57. doi: 10.1021/acs.jpca.5c08602 (PMC12990110; doi:10.1021/acs.jpca.5c08602)
Supplement: Supplementary file 1 [file jp5c08602_si_001.pdf]

SUPPORTING INFORMATION FOR:

Ovalene Photophysics Revisited

Johannes Wega*[a] and Eric Vauthey*[a]

[a] *Department of Physical Chemistry, Quai Ernest Ansermet 30, 1205 Geneva, Switzerland.*

Email: johannes.wega@unige.ch, eric.vauthey@unige.ch

Contents

Page

S1. Comparison between Pyrene and Ovalene Absorption Spectra S2

S2. TD-DFT Calculations S3

S3. Expectations from the Photophysical Model S4
- S3.1. Fluorescence Lifetime S5
- S3.2. Fluorescence Quantum Yield S6
- S3.3. Fluorescence Spectrum S6

S4. Comparison between Calculated and Experimental Emission Spectra S9

S5. Polarization Contributions to Calculated Emission Spectrum S9

S6. Additional Transient Absorption Data S10

S7. Temperature Dependent Emission at 475 nm S10

S8. Global Fit Results S11

S9. Testing the Sensitivity of the Fit S12

S10. References S14

S1. Comparison between Pyrene and Ovalene Absorption Spectra

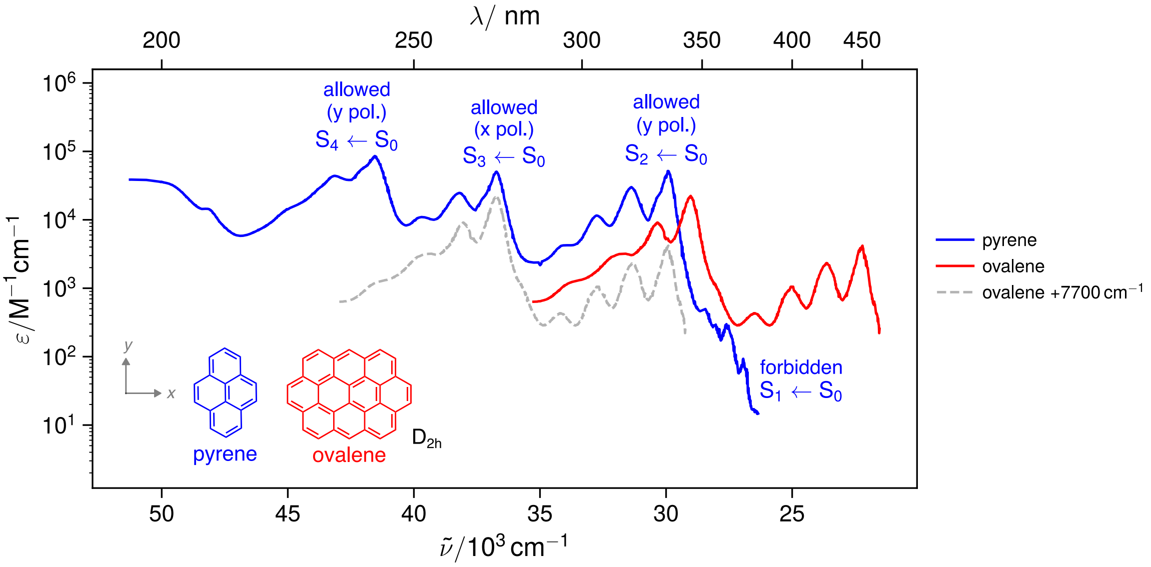

Figure S1: Comparison of the literature absorption spectra of pyrene (blue, solvent: light petrolium) and ovalene (red, solvent: benzene). The spectra were taken from Ref. 8. The observed absorption bands of pyrene are assigned according to the pyrene model (cf. Fig. 1 main text / Refs. 9,10). Shifting the ovalene absorption spectrum by 7700 cm^{-1} / 0.95 eV (dashed gray line) to the blue highlights similarity of the two molecules absorption spectra. The reported literature spectrum of ovalene lacks the forbidden $S_1 \leftarrow S_0$ transition and the first absorption band shown can be assigned to the allowed $S_2 \leftarrow S_0$ transition.

Unfortunately, different molecular coordinate system conventions are used for ovalene[11,12] and pyrene[9,10] in the literature. In this work, we adopt the coordinate system commonly used for ovalene, as shown in the figure above. In contrast, in much of the pyrene literature[9,10] the axis we denote as y is set to the z-axis, while our x-axis corresponds to the y-axis. As a consequence, the symmetry labels of electronic transitions reported for ovalene are different from those reported for pyrene, even though the underlying physics is identical. To facilitate direct comparison with the pyrene literature, the transformation of the molecular axes and the resulting changes in the irreducible representations of the D_{2h} point group are summarized below:

this work → pyrene literature	pyrene literature → this work
$x \rightarrow y$	$x \rightarrow z$
$y \rightarrow z$	$y \rightarrow x$
$z \rightarrow x$	$z \rightarrow y$
$A_g \rightarrow A_g$	$A_g \rightarrow A_g$
$A_u \rightarrow A_u$	$A_u \rightarrow A_u$
$B_{1g} \rightarrow B_{3g}$	$B_{1g} \rightarrow B_{2g}$
$B_{2g} \rightarrow B_{1g}$	$B_{2g} \rightarrow B_{3g}$
$B_{3g} \rightarrow B_{2g}$	$B_{3g} \rightarrow B_{1g}$
$B_{1u} \rightarrow B_{3u}$	$B_{1u} \rightarrow B_{2u}$
$B_{2u} \rightarrow B_{1u}$	$B_{2u} \rightarrow B_{3u}$
$B_{3u} \rightarrow B_{2u}$	$B_{3u} \rightarrow B_{1u}$

S2. TD-DFT Calculations

TABLE **S1**: Comparison of a TD-DFT calculation (CAM-B3LYP/6-31G(d,p)) of ovalene with the pyrene model. The TD-DFT calculation predicts a swapped ordering of the $S_1 \leftarrow S_0$ and $S_2 \leftarrow S_0$ transitions. The columns show: calculated transition, excitation energy (eV), main molecular orbital contributions to the transition, symmetry of the excited state (with the old Platt nomenclature in parenthesis to aid comparison with older literature), oscillator strength, corresponding pyrene model transition, and transition polarization.

TD-DFT	Energy / eV	MO Contributions	Symmetry	Oscillator Strength	Pyrene Model	Polarization
$S_1 \leftarrow S_0$	3.06	LUMO ← HOMO (93 %)	B_{2u} (L_a)	$f = 0.218$	$S_2 \leftarrow S_0$	y
$S_2 \leftarrow S_0$	3.13	LUMO ← HOMO-1 (+51 %)<br>LUMO+1 ← HOMO (−49 %)	B_{3u} (L_b)	$f = 0.0003$	$S_1 \leftarrow S_0$	x (0−0/ a_g vib.)<br>y (b_{1g} vib.)
$S_3 \leftarrow S_0$	3.95	LUMO+2 ← HOMO (+78 %)<br>LUMO ← HOMO-2 (−19 %)	B_{1g}	$f = 0.0000$	-	-
$S_4 \leftarrow S_0$	4.06	LUMO+2 ← HOMO (−14 %)<br>LUMO ← HOMO-2 (+71 %)	B_{1g}	$f = 0.0000$	-	-
$S_5 \leftarrow S_0$	4.23	LUMO ← HOMO-1 (+49 %)<br>LUMO+1 ← HOMO (+51 %)	B_{3u} (B_b)	$f = 1.58$	$S_3 \leftarrow S_0$	x
$S_6 \leftarrow S_0$	4.27	LUMO+3 ← HOMO (73 %)<br>LUMO+2 ← HOMO-1 (+11 %)	A_g	$f = 0.0000$	-	-
$S_7 \leftarrow S_0$	4.45	LUMO+1 ← HOMO-1 (88 %)	B_{2u} (B_a)	$f = 0.48$	$S_4 \leftarrow S_0$	y

S3. Expectations from the Photophysical Model

Before examining the experimental data, we shall first attempt an order-of-magnitude estimate of the expected changes in the experimental observables to be expected from the proposed photophysical model shown in Fig. 2 C (main text).

From the scheme, the rate equations for the concentrations of molecules in the S_2 and S_1 state become:

$$\frac{\mathrm{d}[\mathrm{S}_2]}{\mathrm{d}t} = -(k_{\mathrm{r2}} + k_{\mathrm{nr2}} + k_{\mathrm{IC}})\cdot[\mathrm{S}_2] + k_{-\mathrm{IC}}\cdot[\mathrm{S}_1] \tag{S1}$$

$$\frac{\mathrm{d}[\mathrm{S}_1]}{\mathrm{d}t} = -(k_{\mathrm{r1}} + k_{\mathrm{nr1}} + k_{-\mathrm{IC}})\cdot[\mathrm{S}_1] + k_{\mathrm{IC}}\cdot[\mathrm{S}_2] \tag{S2}$$

where k_{r1} and k_{r2} are the rate constants for radiative deactivation, and k_{nr1} and k_{nr2} are the rate constants for non-radiative deactivation from $S_1 \rightarrow S_0$ and $S_2 \rightarrow S_0$, respectively. The rate constant for internal conversion (IC) from $S_2 \rightarrow S_1$ is k_{IC}, and that for back-IC is $k_{-\mathrm{IC}}$, for which a thermally activated Arrhenius-type expression is proposed due to the expected small energy gap ΔE between S_1 and S_2, i.e. $k_{-\mathrm{IC}} = k_{\mathrm{IC}} e^{-\Delta E/kT}$.

We can estimate the radiative rate constants k_{r1} and k_{r2} by integrating the absorption bands $\varepsilon(\tilde{\nu})$ for the respective transitions using the Strickler–Berg relation:[13–15]

$$k_{\mathrm{rad}} = \frac{2\pi e^2 \nu_0^2}{\epsilon_0 m_{\mathrm{e}} c^3}\left(\frac{n^2+2}{3}\right) n\cdot f = 7.4\times10^{-22}\,\mathrm{s}^{-2}\cdot\frac{n^2+2}{3}\,\nu_0^2\cdot n\cdot f \tag{S3}$$

where n is the refractive index of the solvent, ν_0 is the 0–0 transition frequency, and f is the oscillator strength of the transition:

$$f = \frac{4 m_{\mathrm{e}} c\epsilon_0}{N_A e^2}\ln 10 \int \varepsilon(\tilde{\nu})\,\mathrm{d}\tilde{\nu} = 4.3\times10^{-9}\,\mathrm{M\,cm}^2\cdot\int \varepsilon(\tilde{\nu})\,\mathrm{d}\tilde{\nu} \tag{S4}$$

Analogous to the oscillator strength, we can further estimate the transition dipole moment via:

$$\mu_{\mathrm{TDM}} = 9.584\times10^{-2}\,\mathrm{D}\cdot\mathrm{M}^{-0.5}\,\mathrm{cm}^{0.5}\cdot\sqrt{\frac{2n^2+1}{3n}\int\frac{\varepsilon(\tilde{\nu})}{\tilde{\nu}}\,\mathrm{d}\tilde{\nu}} \tag{S5}$$

This procedure, together with the resulting estimated radiative rate constants, oscillator strengths, and transition dipole moments for both transitions, is summarized in Fig. **S2**.

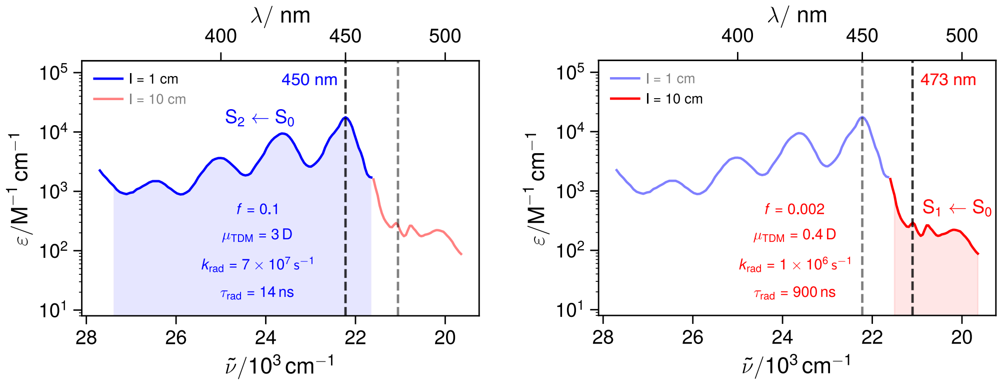

Figure S2: Estimation of the oscillator strength f, transition dipole moment μ_{TDM}, radiative rate constant k_{rad} and natural lifetime τ_{rad} by means of the Strickler-Berg relation of the $S_2 \leftarrow S_0$ transition (blue, left) and $S_1 \leftarrow S_0$ (red, right) transitions of ovalene ($c = 13\,\mu\mathrm{M}$) obtained from absorption spectra in toluene ($n = 1.4941$)[16] measured in a 1 cm and 10 cm cuvette. The employed 0-0 transitions are shown as dashed lines. Using these values an energy gap between the S_2 and S_1 of ΔE =1125cm^{-1} is obtained which is in good agreement with that obtained from the temperature-dependent emission measurements.

As expected, the radiative rate constant for the forbidden $S_1 \leftarrow S_0$ transition is significantly smaller than that of the allowed $S_2 \leftarrow S_0$ transition ($k_{\mathrm{r1}} = 10^6\,\mathrm{s}^{-1}$ vs. $k_{\mathrm{r2}} = 7\times10^7\,\mathrm{s}^{-1}$). Moreover, the estimated radiative lifetime of the S_1 state, $\tau_{\mathrm{rad}} \approx 1\,\mu$s, is in good agreement (in order of magnitude) with the experimental fluorescence lifetime of ovalene measured in an ultracold jet expansion ($\tau_{\mathrm{rad}} = 2.4\,\mu$s)[11], where non-radiative decay and thermal population of the S_2 state are negligible.

Internal conversion typically occurs on a sub-picosecond timescale, i.e. $k_{IC} > 10^{12}\,s^{-1}$, which is further supported by the transient absorption data for ovalene (cf. Fig. 4) and is therefore several orders of magnitude faster than either k_{r1} or k_{r2}. With an energy gap of $\Delta E = 1200\,cm^{-1}$, the thermal population of the S_2 state is approximately 0.3 %, giving $k_{-IC} \approx 3\times10^9\,s^{-1}$, which likewise exceeds the radiative rates by several orders of magnitude. Non-radiative decay of large, rigid polycyclic aromatic hydrocarbons (PAHs) is generally slow.[17] For example, the large structurally similar molecule coronene exhibits $k_{nr} \approx 10^6\,s^{-1}$.[17] Another possible deactivation pathway is intersystem crossing (ISC), but this is expected to be inefficient owing to the $\pi\pi^*$ character of the transitions, weak spin–orbit coupling, and the absence of an El-Sayed–type enhancement.[17] ISC is therefore neglected here for simplicity.

Since both k_{IC} and k_{-IC} exceed all other rate constants by several orders of magnitude, the $S_2 \rightleftharpoons S_1$ manifold rapidly establishes a pre-equilibrium on the sub-picosecond timescale. When exciting into the allowed $S_2 \leftarrow S_0$ transition, the population initially resides entirely in S_2, i.e. $[S_2]_0$. We can then obtain the equilibrium concentration from the equilibrium constant:

$$K = \frac{k_{IC}}{k_{-IC}} = \frac{[S_2]_{eq}}{[S_1]_{eq}} = e^{-\Delta E/kT} \tag{S6}$$

using $[S_2]_{eq} + [S_1]_{eq} = [S_2]_0$, which yields:

$$[S_2]_{eq} = [S_2]_0 \cdot \frac{e^{-\Delta E/kT}}{1+e^{-\Delta E/kT}} \tag{S7}$$

$$[S_1]_{eq} = [S_2]_0 \cdot \frac{1}{1+e^{-\Delta E/kT}} \tag{S8}$$

The expected change in equilibrium populations for an energy gap of $\Delta E = 1200\,cm^{-1}$ upon heating from room-temperature 298 K (25°C) to 423 K (150°C) are shown in Fig. **S3**.

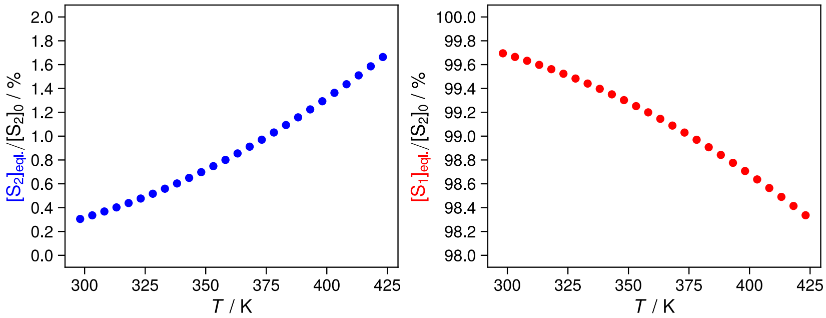

Figure S3: Thermal (pre-)equilibrium populations of the S_2 (left) and S_1 (right) states with an energy gap of $\Delta E = 1200\,cm^{-1}$ when heating from room-temperature 298 K (25°C) to 423 K (150°C).

S3.1 Fluorescence Lifetime

After establishment of the pre-equilibrium, the equilibrium mixture will then decay mono-exponentially via radiative and non-radiative decay from either S_1 or S_2 according to their fractional populations, i.e.:

$$[S_2](t) = [S_2]_0 \cdot \frac{e^{-\Delta E/kT}}{1+e^{-\Delta E/kT}} \cdot e^{-k_{eff}t} \tag{S9}$$

$$[S_1](t) = [S_2]_0 \cdot \frac{1}{1+e^{-\Delta E/kT}} \cdot e^{-k_{eff}t} \tag{S10}$$

$$\text{with:} \qquad k_{eff} = \frac{1}{1+e^{-\Delta E/kT}} \cdot (k_{r1}+k_{nr1}) + \frac{e^{-\Delta E/kT}}{1+e^{-\Delta E/kT}} \cdot (k_{r2}+k_{nr2}) \tag{S11}$$

Figure **S4** A shows an order-of-magnitude estimate of how the fluorescence lifetime of ovalene is expected to change upon heating from room temperature (298 K, 25 °C) to 423 K (150 °C), based on the parameters estimated in Table **S2**. Although the thermal population of S_2 increases by just over 1% across this temperature range, the fluorescence lifetime is expected to decrease by more than 100 ns. This is because a slightly higher S_2 population, with its much larger k_{r2}, leads to faster radiative decay and therefore a shorter overall lifetime.

TABLE **S2**: Estimated photophysical parameters for the computed order-of-magnitude estimates of the changes of experimental properties computed in this section.

parameter	estimated value
k_{r1}/s^{-1}	10^6
k_{r2}/s^{-1}	7×10^7
$k_{nr1,\,nr2}/s^{-1}$	10^6
$\Delta E/cm^{-1}$	1200

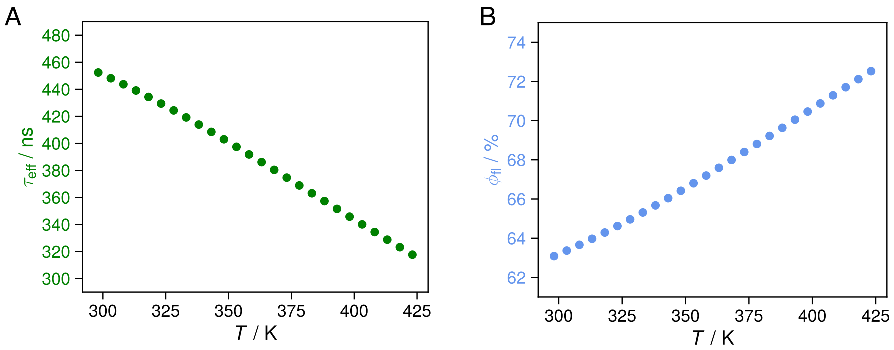

Figure S4: Order of magnitude estimate in the change of the fluorescence lifetime (**A**) and fluorescence quantum yield (**B**) of ovalene when heating from room-temperature 298 K (25°C) to 423 K (150°C) taking an S_2-S_1 energy gap of $\Delta E = 1200\,\mathrm{cm}^{-1}$.

S3.2 Fluorescence Quantum Yield

The fluorescence quantum yield ϕ_{fl} is defined as the number of photons emitted relative to the number of photons absorbed by the molecule. If excitation occurs in the S_2 state, the initial concentration of absorbed photons is $[S_2]_0$, and the fluorescence quantum yield can be written as:

$$\phi_{fl} = \frac{[h\nu]_{tot}}{[S_2]_0} \tag{S12}$$

where $[h\nu]_{tot} = [h\nu]_1 + [h\nu]_2$ is the total concentration of photons emitted from the S_1 and S_2 states. The concentrations of photons emitted from the S_1 and S_2 states are

$$[h\nu]_1 = k_{r1}\cdot\int_0^\infty [S_1](t)\,dt = [S_2]_0\cdot\frac{1}{1+e^{-\Delta E/kT}}\cdot\frac{k_{r1}}{k_{eff}(T)} \tag{S13}$$

$$[h\nu]_2 = k_{r2}\cdot\int_0^\infty [S_2](t)\,dt = [S_2]_0\cdot\frac{e^{-\Delta E/kT}}{1+e^{-\Delta E/kT}}\cdot\frac{k_{r2}}{k_{eff}(T)} \tag{S14}$$

Using these relations in Eq. S12, the fluorescence quantum yield becomes:

$$\phi_{fl} = \frac{k_{r1}}{k_{r1}+k_{nr1}+e^{-\Delta E/kT}\cdot(k_{r2}+k_{nr2})} + \frac{k_{r2}\cdot e^{-\Delta E/kT}}{k_{r1}+k_{nr1}+e^{-\Delta E/kT}\cdot(k_{r2}+k_{nr2})} \tag{S15}$$

As expected for conventional molecules with a large S_2-S_1 gap, i.e. $\Delta E \to \infty$ and therefore $e^{-\Delta E/kT} \to 0$, the fluorescence quantum yield reduces to $\phi_{fl} \to k_{r1}/(k_{r1}+k_{nr1})$, i.e. the conventional fluorescence quantum yield expression for the S_1 state. Figure **S4** B shows the estimated change in the fluorescence quantum yield of ovalene upon heating from room temperature 298 K, (25 °C) to 423 K (150 °C). As the thermal population of S_2 increases, the effective radiative rate constant also increases, leading to a higher overall concentration of emitted photons as less excited states decay non-radiatively. This results in an increase of the fluorescence quantum yield by approximately 10% over this temperature range.

S3.3 Fluorescence Spectrum

The total fluorescence spectrum is given as the sum of the individual fluorescence spectra for the $S_1 \to S_0$ and $S_2 \to S_0$ transitions:

$$F(\lambda, T) = F_1(\lambda, T) + F_2(\lambda, T) \tag{S16}$$

The individual fluorescence spectra for both transitions can be obtained from the number of emitted photons from each state:

$$F_1(\lambda, T) = c \cdot g_1(\lambda) \cdot k_{r1} \cdot [h\nu]_1 = c \cdot g_1(\lambda) \cdot k_{r1} \cdot [S_2]_0 \cdot \frac{1}{1+e^{-\Delta E/kT}} \cdot \frac{k_{r1}}{k_{eff}(T)} \tag{S17}$$

$$F_2(\lambda, T) = c \cdot g_2(\lambda) \cdot k_{r2} \cdot [h\nu]_2 = c \cdot g_2(\lambda) \cdot k_{r2} \cdot [S_2]_0 \cdot \frac{e^{-\Delta E/kT}}{1+e^{-\Delta E/kT}} \cdot \frac{k_{r2}}{k_{eff}(T)} \tag{S18}$$

where $g_1(\lambda)$ and $g_2(\lambda)$ are the area-normalized line-shape functions of the $S_1 \rightarrow S_0$ and $S_2 \rightarrow S_0$ transitions, respectively, and c is a conversion factor for emitted photons to fluorescence intensity. Using these relations together with the expression for k_{eff}, the total fluorescence spectrum becomes:

$$F(\lambda, T) = c \cdot [S_2]_0 \cdot \left[g_1(\lambda) \cdot \frac{k_{r1}}{k_{r1} + k_{nr1} + e^{-\Delta E/kT} \cdot (k_{r2} + k_{nr2})} + g_2(\lambda) \cdot \frac{k_{r2} \cdot e^{-\Delta E/kT}}{k_{r1} + k_{nr1} + e^{-\Delta E/kT} \cdot (k_{r2} + k_{nr2})} \right] \tag{S19}$$

In the limit of very large energy gaps, i.e., $e^{-\Delta E/kT} \rightarrow 0$, the fluorescence spectrum reduces to the emission spectrum of the $S_1 \rightarrow S_0$ transition, proportional to the fluorescence quantum yield ϕ_{fl1} of the S_1 state and the line-shape function of the transition:

$$F(\lambda, T) \rightarrow c \cdot [S_2]_0 \cdot g_1(\lambda) \cdot \underbrace{\frac{k_{r1}}{k_{r1} + k_{nr1}}}_{=\phi_{fl1}} \tag{S20}$$

as expected.

To simulate the expected change of the fluorescence with temperature, the lineshape functions of the two transitions are needed. For the $S_1 \rightarrow S_0$ transition, we take the lineshape function $g_1(\lambda)$ as the experimental emission spectrum at 133 K (−140 °C) in toluene reported by Kropp and Stanley[18] (cf. Fig. **S5**), as the thermal population of the S_2 state is effectively zero at this temperature. For the lineshape function of the $S_2 \rightarrow S_0$ transition, we take the mirror image of the $S_2 \leftarrow S_0$ absorption band with a small Stokes shift of 120 cm^{-1}, typical for allowed transitions in PAHs.

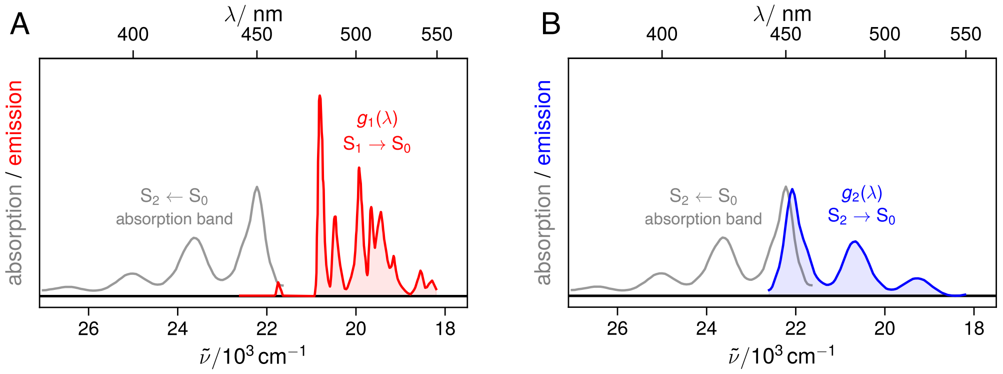

Figure S5: **A**: $S_1 \rightarrow S_0$ lineshape taken as the emission spectrum of ovalene at −140 °C in toluene reported in Ref. 18. **B**: $S_2 \rightarrow S_0$ lineshape taken as the mirror image of the $S_2 \leftarrow S_0$ absorption band with a Stokes shift of 120 cm^{-1}.

With the lineshape functions at hand, we can now qualitatively simulate the expected changes in the fluorescence spectrum upon heating from room temperature (298 K, 25 °C) to 423 K (150 °C). The results of these simulations are shown in Fig. **S6**. As can be seen, upon heating the emission bands of the $S_2 \rightarrow S_0$ transition grow significantly, while those of the $S_1 \rightarrow S_0$ transition decrease in intensity.

Fig. **S7** shows the same simulation, but using the energy gap of $\Delta E = 400\,\text{cm}^{-1}$ reported by Kropp and Stanley.[18] As the simulation indicates, such a small gap would lead to fluorescence spectra dominated by the $S_2 \rightarrow S_0$ emission already at room temperature, in clear contradiction to the experimental observations.

To obtain the S_1–S_2 energy gap experimentally, we analyze the fluorescence intensity at a wavelength where emission arises predominantly from the $S_2 \rightarrow S_0$ transition, i.e. where $g_1(\lambda) = 0$. In this case, Eq. S20 simplifies to

$$F_2(\lambda, T) = c\,[S_2]_0\,g_2(\lambda)\,\frac{k_{r2}\,e^{-\Delta E/kT}}{k_{r1} + k_{nr1} + e^{-\Delta E/kT}(k_{r2} + k_{nr2})} = C(\lambda)\,\frac{e^{-\Delta E/kT}}{1+\xi\,e^{-\Delta E/kT}}, \tag{S21}$$

with

$$\xi = \frac{k_{r2}+k_{nr2}}{k_{r1}+k_{nr1}}, \qquad C(\lambda) = c\,[S_2]_0 \cdot \frac{k_{r1}}{k_{r2}+k_{nr2}} \cdot g_2(\lambda)$$

Here, $C(\lambda)$ is an emission wavelength dependent constant. Given that for $\Delta E = 1200\,\mathrm{cm}^{-1}$ one obtains $e^{-\Delta E/kT} \sim 10^{-3}$ in the temperature range of interest and $\xi \approx 40$ (cf. Tab. **S2**), we have $1 \gg \xi\, e^{-\Delta E/kT}$. The denominator can therefore be expanded as a geometric series, i.e.

$$\frac{1}{1+\xi e^{-\Delta E/kT}} \approx 1 - \xi e^{-\Delta E/kT}$$

which shows that the S_2 fluorescence intensity follows, to a good approximation, a simple Boltzmann dependence:

$$F_2(\lambda, T) \approx C(\lambda)\, e^{-\Delta E/kT} \tag{S22}$$

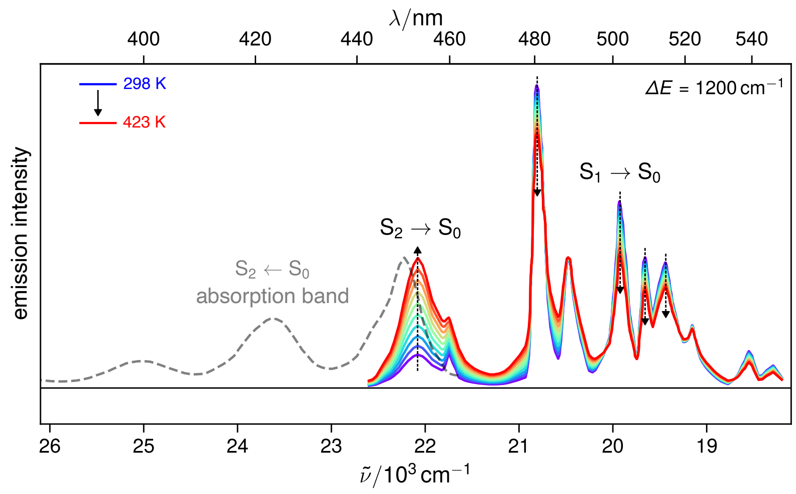

Figure S6: Simulated change in the fluorescence spectrum of ovalene when heating from room temperature (298 K, 25 °C) to 423 K (150 °C), assuming an S_2–S_1 energy gap of $\Delta E = 1200\,\mathrm{cm}^{-1}$.

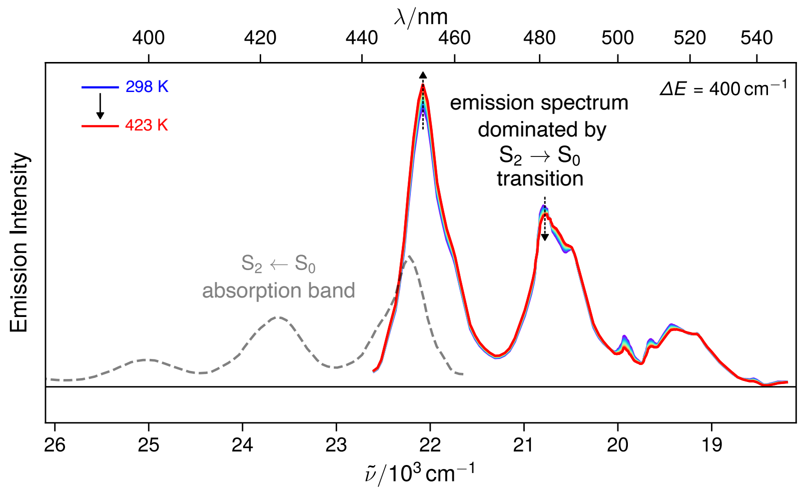

Figure S7: Simulated change in the fluorescence spectrum of ovalene when heating from room temperature (298 K, 25 °C) to 423 K (150 °C), assuming an S_2–S_1 energy gap of $\Delta E = 400\,\mathrm{cm}^{-1}$.

S4. Comparison between Calculated and Experimental Emission Spectra

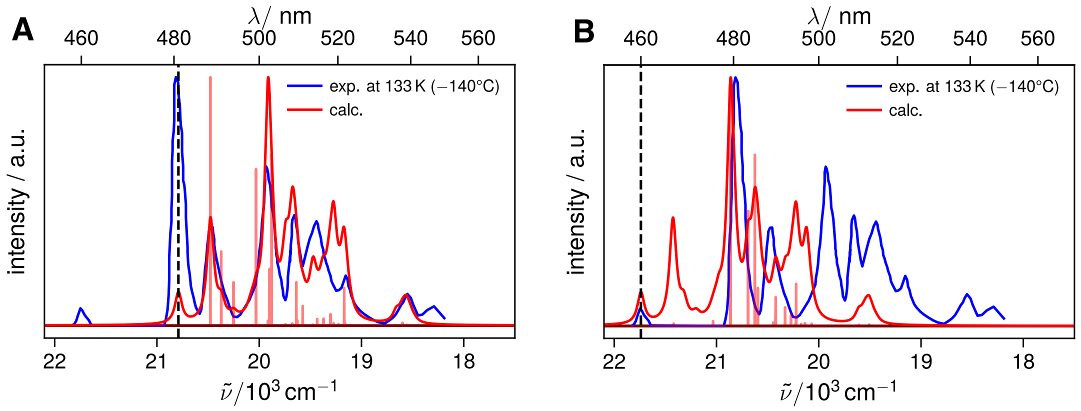

Figure S8: Comparison of the experimental $S_1 \longrightarrow S_0$ emission spectrum of ovalene at −140 °C in toluene (blue line) reported in Ref. 18 with the theoretically calculated TD-DFT Herzberg-Teller spectrum by Weber et al.[12] (red sticks). The transition energies and intensities were taken from Table S3 of Ref. 12. The solid red line corresponds to a broadened spectrum obtained by centering a Lorentzian with FWHM = 80 cm^{-1} on each vibronic transition. **A:** Comparison when the 0–0 transition is placed at 480 nm, consistent with an energy gap of $\Delta E = 1200$ cm^{-1}. **B:** Comparison when the 0–0 transition is placed at the historic p-band at 460 nm, corresponding to an energy gap of 400 cm^{-1}. The 0–0 transitions are indicated by dashed black lines. The experimental peak at 460 nm thus likely arises from an experimental artifact, e.g., a Raman peak.

S5. Polarization Contributions to Calculated Emission Spectrum

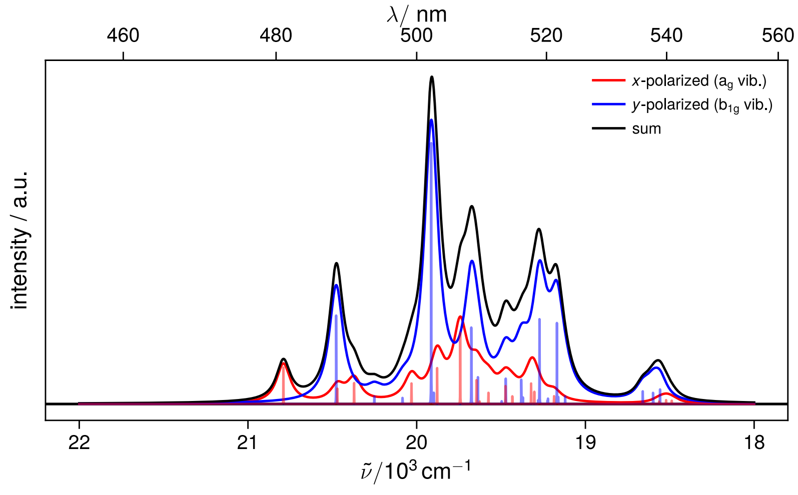

Figure S9: TD-DFT–calculated Herzberg–Teller vibronic $S_1 \longrightarrow S_0$ emission spectrum of ovalene from Weber et al.[12], showing both x-polarized (a_g, red sticks) and y-polarized (b_{1g}, blue sticks) transitions. The transition energies and intensities were taken from Table S3 of Ref. 12. The solid lines represent the broadened spectra obtained by centering a Lorentzian with a FWHM of 80 cm^{-1} on each vibronic transition.

S6. Additional Transient Absorption Data

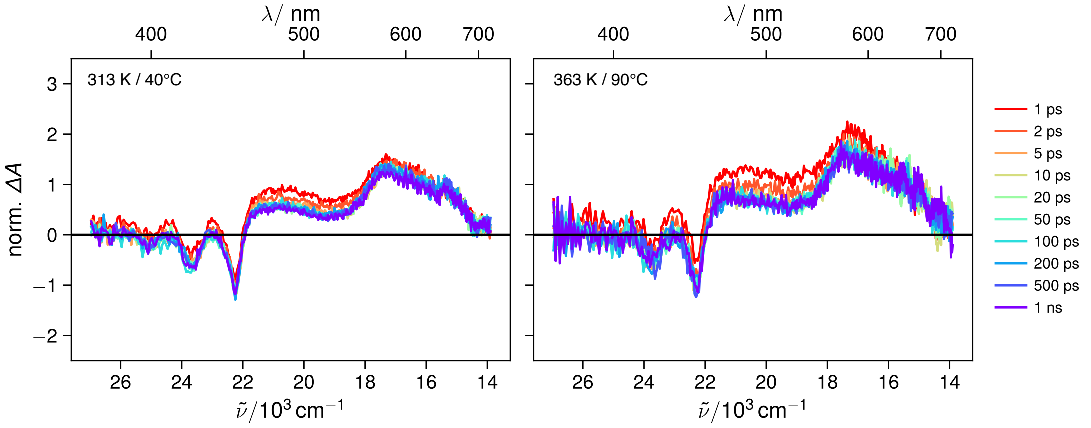

Figure S10: Spectral slices of the transient absorption spectrum of ovalene in toluene at indicated delays after excitation in the $S_3 \leftarrow S_0$ transition at 340 nm at 313 K (40°C, left) and 363 K (90°C, right) . All spectra were normalized to the integral over the ground-state bleach region. No significant dynamics or spectral evolution are detected, and the fact that the ESA band is already present at the earliest delay times indicates that internal conversion occurs within the instrument response function (< 1 ps).

S7. Temperature Dependent Emission at 475 nm

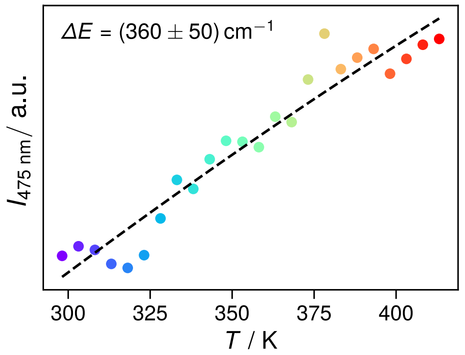

Figure S11: Temperature-dependent steady-state emission intensity at 475 nm (dots) and fit to Eq. S22 (dashed black line), yielding an apparent gap of $\Delta E \approx 400\,\mathrm{cm}^{-1}$ as was done in the study of Kropp and Stanley.[18] However, as described in detail in the main text, fitting the 475 nm intensity to Eq. S22 is not physically meaningful, because emission at this wavelength contains a substantial contribution from the $S_1 \rightarrow S_0$ transition rather than exclusively emission from $S_2 \rightarrow S_0$.

S8. Global Fit Results

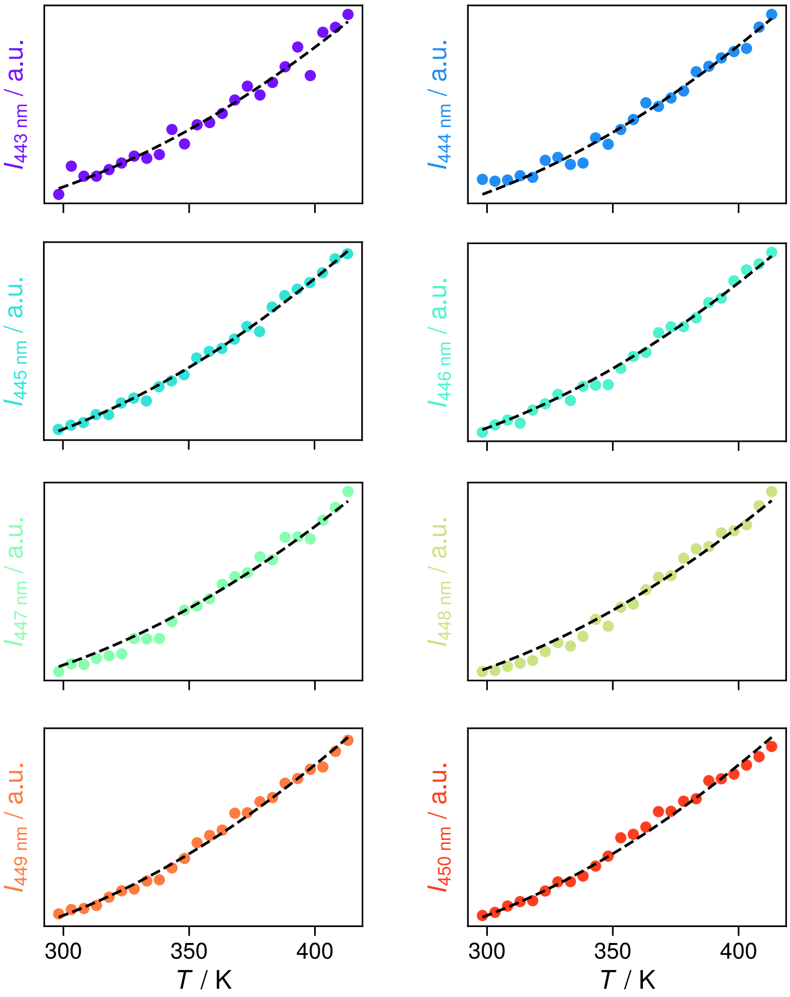

Figure S12: A few additional intensity vs. temperature traces from the $S_2 \rightarrow S_0$ emission band of the temperature-dependent emission spectrum of Ovalene shown in Fig. 4. The dashed black lines represent the result of a gobal fit to Eq. S22 with the same S_2–S_1 energy gap of $\Delta E = (1170 \pm 100)\,\mathrm{cm}^{-1}$. In total 38 different wavelength points inside the $S_2 \rightarrow S_0$ emission band were included in the global fit.

S9. Testing the Sensitivity of the Fit

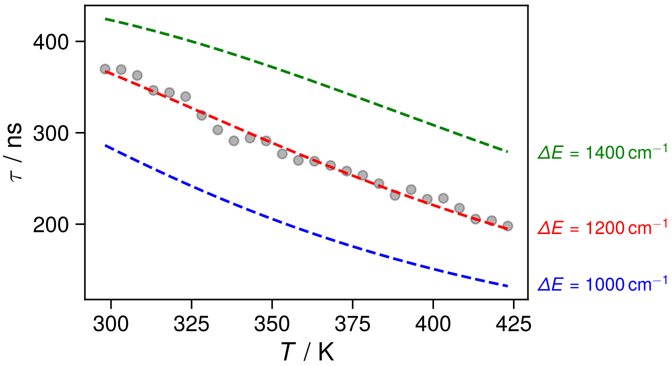

Figure S13: Best non-linear least-squares fit of Eq.S11 (Eq.3 main text) with $\tau(T) = 1/k_{\mathrm{eff}}(T)$ to the measured temperature dependence of the fluorescence lifetime of ovalene in a PVB film using different ΔE values and τ_1 and τ_2 as adjustable parameters.

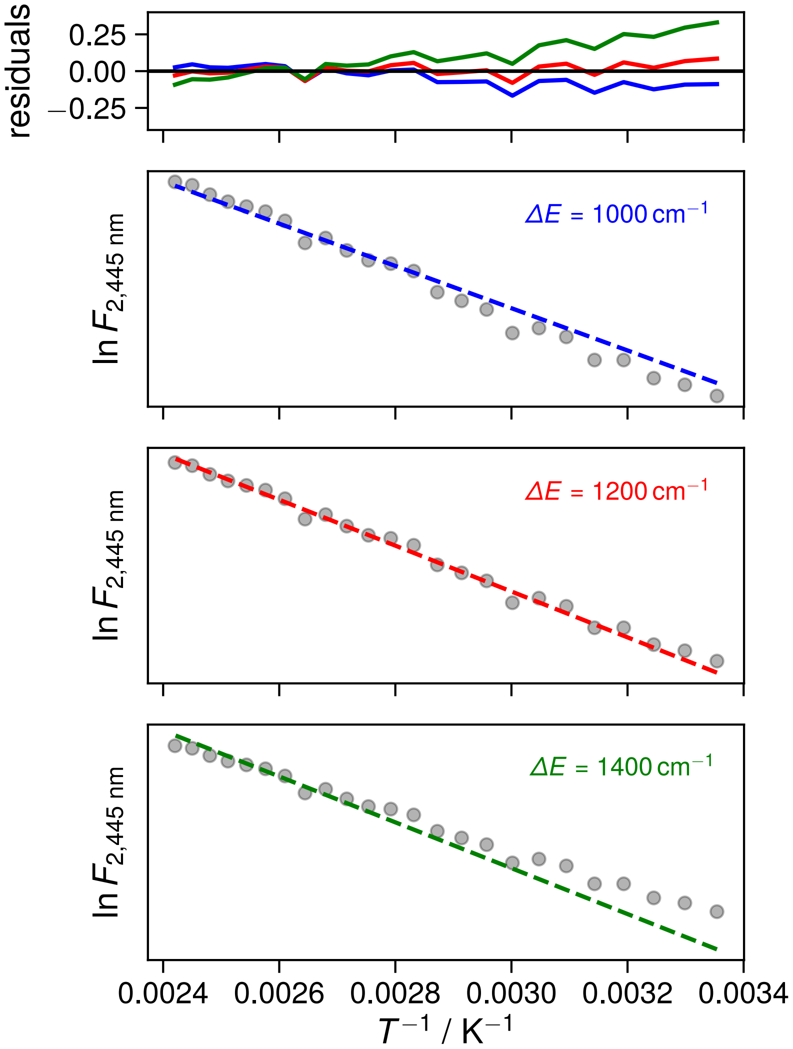

Figure S14: Best fit of Eq.S22 (Eq. 4 in main text) to the emission intensity at 445 nm in the $S_2 \rightarrow S_0$ band with different ΔE values and C as adjustable parameter.

S10. References

[1] Gardecki, J.; Maroncelli, M. Set of secondary emission standards for calibration of the spectral responsivity in emission spectroscopy. *App. Spec.* **1998**, *52*, 1179–1189.

[2] Walfort, B.; Gartmann, N.; Afshani, J.; Rosspeintner, A.; Hagemann, H. Effect of Excitation Wavelength (Blue vs Near UV) and Dopant Concentrations on Afterglow and Fast Decay of Persistent Phosphor SrAl2O4: Eu2+, Dy3+. *J. Rare Earths* **2022**, *40*, 1022–1028.

[3] Zhao, L.; Lustres, J. L. P.; Farztdinov, V.; Ernsting, N. P. Femtosecond Fluorescence Spectroscopy by Upconversion with Tilted Gate Pulses. *Phys. Chem. Chem. Phys.* **2005**, *7*, 1716–1725.

[4] Beckwith, J. S. Investigating Photoinduced Charge-transfer Products Using Ultrafast Spectroscopy. Ph.D. thesis, University of Geneva, 2019.

[5] Lang, B. Photometrics of ultrafast and fast broadband electronic transient absorption spectroscopy: State of the art. *Rev. Sci. Instrum.* **2018**, *89*.

[6] Beckwith, J. S.; Rumble, C. A.; Vauthey, E. Data analysis in transient electronic spectroscopy – an experimentalist's view. *Int. Rev. Phys. Chem.* **2020**, *39*, 135–216.

[7] Frisch, M. J. et al. Gaussian~16 Revision C.01. 2016; Gaussian Inc. Wallingford CT.

[8] Perkampus, H.; Sandeman, I.; Timmons, C. *UV Atlas of Organic Compounds*; Butterworth/Verlag Chemie: London/Weinheim, 1966.

[9] Crawford, A. G.; Dwyer, A. D.; Liu, Z.; Steffen, A.; Beeby, A.; Palsson, L.-O.; Tozer, D. J.; Marder, T. B. Experimental and theoretical studies of the photophysical properties of 2-and 2, 7-functionalized pyrene derivatives. *J. Am. Chem. Soc.* **2011**, *133*, 13349–13362.

[10] Michl, J.; Thulstrup, E. *Spectroscopy with Polarized Light: Solute Alignment by Photoselection, in Liquid Crystals, Polymers, and Membranes*; VCH, 1995.

[11] Amirav, A.; Even, U.; Jortner, J. Excited-state dynamics of the isolated ultracold ovalene molecule. *J. Chem. Phys.* **1981**, *74*, 3745–3756.

[12] Weber, I.; Langner, J.; Witek, H. A.; Lee, Y.-P. Electronic Spectroscopy of Ovalene: Reassignment of the S_2 (B_{3u})–S_0 (A_g) Transition. *J. Phys. Chem. Lett.* **2024**, *15*, 10696–10702.

[13] Strickler, S.; Berg, R. A.; others Relationship between absorption intensity and fluorescence lifetime of molecules. *J. Chem. Phys.* **1962**, *37*, 814.

[14] Delaire, J.; Piard, J.; Méallet-Renault, R.; Clavier, G. *Photophysique et photochimie: des fondements aux applications*; EDP Sciences, 2016.

[15] Angulo, G.; Grampp, G.; Rosspeintner, A. Recalling the appropriate representation of electronic spectra. *Spectrochim. Acta A* **2006**, *65*, 727–731.

[16] Marcus, Y. *The Properties of Solvents*; The Properties of Solvents Bd. 1; Wiley: Chichester, 1998.

[17] Birks, J. B. *Photophysics of Aromatic Molecules*; Wiley; Wiley: New York, 1970.

[18] Kropp, J. L.; Stanley, C. C. The temperature dependence of ovalene fluorescence. *Chem. Phys. Lett.* **1971**, *9*, 534–538.
